# Thermodynamics and kinetics of DNA nanotube polymerization from single-filament measurements†
†Electronic supplementary information (ESI) available. See DOI: 10.1039/c3sc53331j



**DOI:** 10.1039/c3sc53331j

**Published:** 2015-02-20

**Authors:** Rizal F. Hariadi, Bernard Yurke, Erik Winfree

**Affiliations:** a Applied Physics , California Institute of Technology , Pasadena , California 91125 , USA; b Materials Science and Engineering Department and Electrical and Computer Engineering Department , Boise State University , Boise , Idaho 83725 , USA; c Bioengineering , California Institute of Technology , Pasadena , California 91125 , USA . Email: winfree@caltech.edu

## Abstract

DNA nanotubes provide a programmable architecture for molecular self-assembly and can serve as model systems for one-dimensional biomolecular assemblies. While a variety of DNA nanotubes have been synthesized and employed as models for natural biopolymers, an extensive investigation of DNA nanotube kinetics and thermodynamics has been lacking. Using total internal reflection microscopy, DNA nanotube polymerization was monitored in real time at the single filament level over a wide range of free monomer concentrations and temperatures. The measured polymerization rates were subjected to a global nonlinear fit based on polymerization theory in order to simultaneously extract kinetic and thermodynamic parameters. For the DNA nanotubes used in this study, the association rate constant is (5.99 ± 0.15) × 10^5^ M^–1^ s^–1^, the enthalpy is 87.9 ± 2.0 kcal mol^–1^, and the entropy is 0.252 ± 0.006 kcal mol^–1^ K^–1^. The qualitative and quantitative similarities between the kinetics of DNA nanotubes, actin filaments, and microtubules polymerization highlight the prospect of building complex dynamic systems from DNA molecules inspired by biological architecture.

## Abstract 2

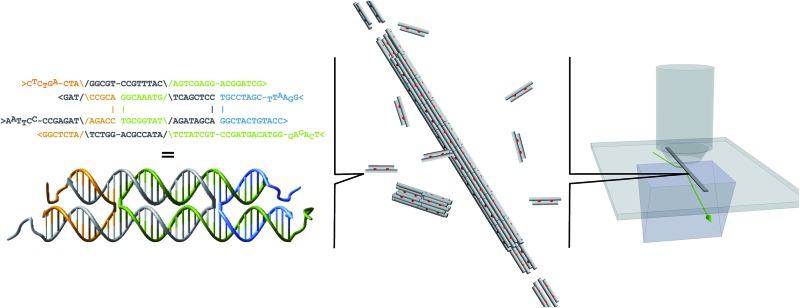
Single-filament measurement of the thermodynamic and kinetic parameters of DNA nanotube assembly supports a polymerization/depolymerization model sharing common features with cytoskeletal polymer models.

## Introduction

The design and construction of collective behaviors out of rigorously-characterized molecular elements that rival cellular systems is a challenge at the interface of biology, chemistry, physics, and computer science. Molecular biology provides many proofs of principle for how chemistry can be used to self-organize matter.^1^ As an example, the cytoskeleton is a system of intracellular biopolymers that evaluates its environment to assemble and disassemble at the right time and the right place within cells. Interactions between the cytoskeleton, molecular motors, and signaling proteins give rise to self-organized intracellular structure^2^ and motility,^3^ direct the growth of tissues,^4^ and guide the movement of organisms.^5,6^

DNA nanotubes^7,8^ have been proposed as a promising candidate material for *de novo* engineering of an artificial cytoskeleton.^9^ A successful demonstration of an artificial cytoskeleton will recapitulate structural, dynamic, force generation, and assembly control aspects of the biological cytoskeleton. Toward that goal, we must understand the design principles of dynamic tubular architectures and develop an accurate physical model of how monomers can assemble and disassemble tubular structures as they respond to information in the environment.

**Fig. 1:**
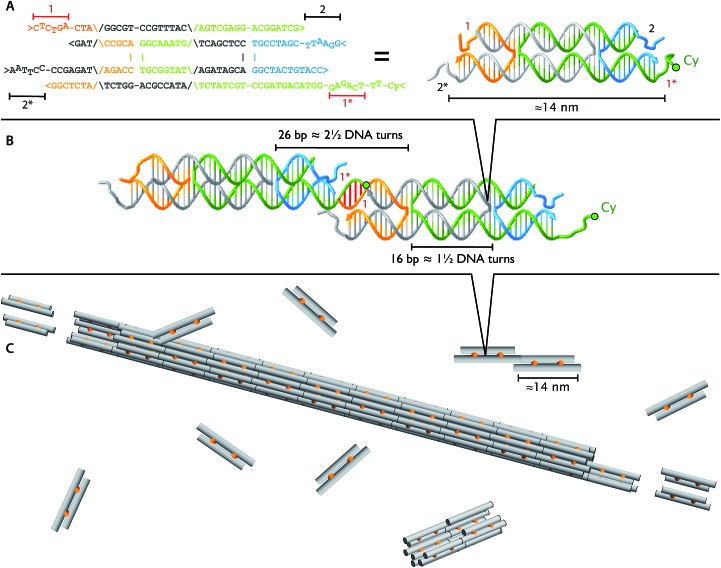
*Identical DNA tiles self-assemble into DNA nanotubes*. The four DNA strands form two DNA helices that are joined by two cross-over points (A and B), depicted as orange connectors in the cartoon (C). Each tile is roughly 2 × 4 × 14 nm and has four single stranded DNA sticky ends (1, 1*, 2, and 2*). The four sticky ends are designed such that 1 is complementary to 1* (red sticky end pairs) and 2 is complementary to 2* (black sticky end pairs). In the dimer picture (B), the left monomer appears thinner due to 36° rotation along the horizontal axis to match the minor and major groove between the ends of two monomers at the sticky end region. For fluorescence imaging, we labeled one of the DNA strands with Cy3 or Cy5 fluorophore (green circles; Table 1).

In structural DNA nanotechnology, synthetic oligonucleotides can be engineered to form a small DNA complex, called a DNA tile, that can polymerize to form larger structures using the specificity of Watson–Crick hybridization.^7,10–16^ In this paper, we use DNA tile and DNA monomer interchangeably. DNA nanotubes provide a simple example of how a long one-dimensional crystalline structure can arise from the interaction between DNA tiles. Fig. 1 shows a DNA tile that possesses 4 short single-stranded regions, known as sticky ends, that serve as binding domains. The sticky end arrangement, in addition to the constraint provided by the biophysical properties of the DNA double helix, directs the interaction of DNA tiles to form tubular DNA structures with a range of circumferences whose distribution is determined by the thermodynamics and the kinetics of the DNA nanotube assembly process.

**Table 1:** DNA sequences for a single-monomer-type DNA nanotube. For the fluorophore-labeled strands, we inserted two additional T's between the fluorophore and NB-3 sequence as a spacer to minimize any potential side effect of having the Cy3 or Cy5 fluorophore at the end of a sticky end

Name	Sequence
NB-1	5′-CTCTGA-CTACCGCACCAGAATCTCGG-3′
NB-2	5′-AATTCC-CCGAGATTCTGGACGCCATAAGATAGCACCTCGACTCATTTGCCTGCGGTAG-3′
NB-3	5′-TCAGAG-GGTACAGTAGCCTGCTATCTTATGGCGTGGCAAATGAGTCGAGGACGGATCG-3′
NB-3-Cy3	5′-Cy3-TT-TCAGAG-GGTACAGTAGCCTGCTATCTTATGGCGTGGCAAATGAGTCGAGGACGGATCG-3′
NB-3-Cy5	5′-Cy5-TT-TCAGAG-GGTACAGTAGCCTGCTATCTTATGGCGTGGCAAATGAGTCGAGGACGGATCG-3′
NB-4	5′-GGAATT-CGATCCGTGGCTACTGTACC-3′

The first challenge for *de novo* construction of an artificial cytoskeleton is constructing a long and rigid polymer out of artificial non-covalently-bound subunits. DNA nanotubes satisfy the length and rigidity criteria. Structurally, the DNA nanotubes used in this work are cooperative polymers that are multiple monomers wide. The cooperativity has two important consequences. First, the tubular organization of DNA tiles along the longitudinal axis of a DNA nanotube gives rise to a long persistence length, *ξ*tubep ∼ 20 μm,^7,17^ which is comparable to the measured persistence length of actin filaments, *ξ*actinp = 6–25 μm.^18–20^ Second, formation of cooperative polymers at reaction conditions where spontaneous nucleation is rare, gives rise to long polymers. The mean length of the DNA nanotubes used in this study is on the order of 5 μm for standard assembly conditions. Each DNA tile added to the tip of a growing nanotube interacts with two neighbors, whereas most of the collisions between DNA tiles in solution result in contact with only one neighbor. As a result, there is a high kinetic barrier associated with nucleation whereas elongation proceeds without significant barrier.^13,21^ Therefore, to study elongation rates, it is useful to circumvent nucleation by providing a seed – either an appropriately designed DNA origami onto which tiles can readily assemble^22,23^ or, as in this work, pre-formed DNA nanotubes that serve as nuclei for further self-assembly. This kind of kinetic barrier is also common for biological polymers, such as actin filaments and microtubules.^24^

Engineering a dynamic DNA nanotube analog of the cytoskeleton requires an accurate polymerization model for DNA nanotubes. In the literature, there are two classes of models that are relevant to the polymerization of DNA nanotubes, namely the kinetic Tile Assembly Model (kTAM) developed for DNA tile assembly and the polymerization theory developed for biopolymers. In the DNA self-assembly literature, the kTAM considers growth by tile self-assembly to be a second order chemical reaction between crystals and monomers, which will be described in more detail in results and discussion. The kTAM has been used to guide the design and provide a deeper understanding of algorithmic self-assemblies of DNA tile sets with various levels of complexity.^13,22,25–28^ In the biophysics literature, there is a different class of polymerization models for active one-dimensional polymers that couple their polymerization with fuel consumption reactions in the form of nucleotide hydrolysis.^29,30^ Since DNA nanotubes are passive one-dimensional polymers that comprise a single monomer type, these two classes of models are essentially equivalent when applied to existing DNA nanotubes if one ignores the active aspects of the biophysics model and the multiple tile aspects of the kTAM. We will refer to these restriction of both models as the ‘common core model’ for nanotube polymerization.

Despite the success of the kTAM for guiding the design of complex DNA self-assembly systems, the theoretical framework and its assumptions have not been tested experimentally in detail. The rigorous testing of DNA nanotube polymerization theory requires assays that can determine not only the concentration of free DNA tile monomers, but also the number of nanotubes at any given time, and the rate of growth, without experimentally confounding effects, such as excessive spontaneous nucleation or the presence of tube bundles. Early studies of DNA ribbons^13^ used bulk UV absorbance data, in combination with static atomic force microscopy (AFM) assays, to measure the concentration of DNA tiles free in solution, and thereby to infer the kinetics of incorporation into ribbon assemblies. Interpreting bulk data is complicated, because polymer growth kinetics depends not only on free monomer concentration, but also on the size distribution of supramolecular assemblies and on the number of such assemblies. This information cannot be accurately measured in bulk UV absorbance assays and must be inferred indirectly, thus, introducing large uncertainties into the analysis. Recently, Evans *et al.* used a single-molecule AFM movie to validate some of the kTAM assumptions for polymerization on mica surface.^31^ Despite their rigorous analysis, the interaction between DNA tiles and mica surfaces complicated their measurements and limited their ability to determine quantitative measurements of the rate constants and free monomer concentrations.

In this work, we adopted the standard assay in biopolymer research, namely time-lapse light microscopy.^32–34^ The power of single-filament cinematography has enabled the continuous observation of non-equilibrium polymers. To minimize background fluorescence from the sea of unlabeled monomers in solution, fluorescent polymers must be excited either with an evanescent wave by total internal reflection fluorescence (TIRF) microscopy^35–37^ or by confocal illumination.^38^

Here, we report the application of TIRF microscopy to the study of the polymerization of self-assembled DNA structures. From a set of polymerization movies at a wide range of tile concentrations and reaction temperatures, we were able to measure both the kinetic and thermodynamic parameters of DNA nanotube assembly. The experimental results are consistent with the common core model for nanotube polymerization; further, they provide reliable direct measurements of key thermodynamic and kinetic parameters for DNA tile self-assembly and thus help resolve inconstancies among values that were previously measured by indirect means.

## Materials and methods

### Total internal reflection fluorescence microscope

#### Optics

**Fig. 2:**
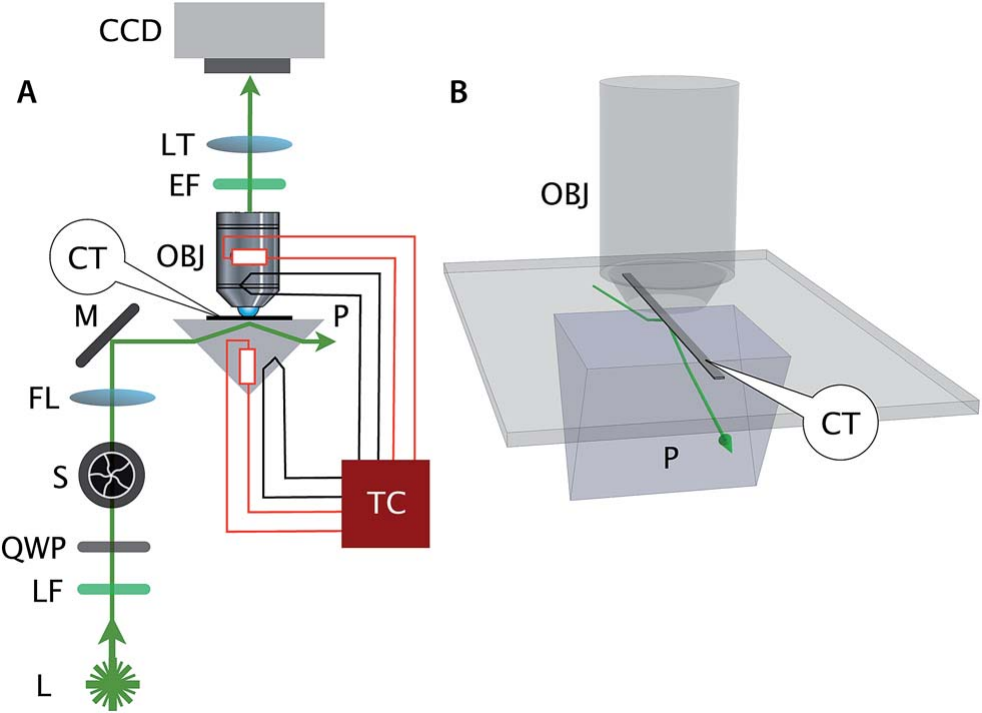
*The schematic of the prism based TIRF microscope*. (A) *L* = 532 nm 25 mW laser; LF = laser filter; QWP = quarter wave plate; S = mechanical shutter; FL = 15 cm focusing lens; M = mirror; P = temperature controlled right angle prism; OBJ = temperature controlled 1.2 NA water immersion objective; EF = emission filter; LT = 20 cm tube lens; CCD = electron multiplier Charged Coupled Device; TC = temperature control. (B) Glass capillary flow chamber (CT) for the polymerization assay. In our upright microscope setup, the flow chamber (mounted on a glass slide) is sandwiched between the prism and the objective. We use immersion oil to optically couple and mount the bottom surface of the 1 mm thick microscope slide with the glass prism. A thin layer of epoxy was used to optically couple the glass capillary chamber to a microscope slide.

The polymerization movies were acquired with a custom-built prism-based total internal reflection fluorescence (TIRF) upright microscope (Fig. 2). A solid-state green laser (GCL-025, 25 mW, CrystaLaser) equipped with an adjustable power supply (CL2005, CrystaLaser) provides the 532 nm excitation light. The beam was filtered with a Z532/10× laser filter (Chroma). The filtered excitation beam passed through a quarter-wave plate (Thorlabs) to produce a circularly polarized beam, which effectively has uniform polarization to counter the orientation-dependent fluorescence of Cy3. A shutter is used to block the light path between snapshots, to reduce photobleaching. Two mirrors (Thorlabs, not pictured in the drawing) were used to guide the illumination beam to the field of view below the objective. Another mirror (Thorlabs) and a 15 cm focusing lens (CVI Melles Griot) steered the excitation beam onto a Suprasil 1 right-angle prism (CVI Melles Griot) at approximately 0° from the horizon (*i.e.*, entering at an ∼45° angle to the prism surface) to produce a weakly focused illumination spot on the microscope slide. We calculated that the incident angle between the incoming laser and the normal vector of the microscope slide (∼77°) is sufficiently larger than the critical angle (∼63°) for an evanescent wave to occur at the interface between glass and liquid, where the sample and focal plane of the objective are located.

In our experiment, DNA tiles and DNA nanotubes resided inside a rectangular glass capillary tube that was situated between the prism and objective as shown in Fig. 2. The emitted photons were captured by a 60× 1.2 NA water immersion objective (Nikon) and focused to the electron multiplier CCD camera (C9100-02, Hamamatsu) by a 20 cm tube lens (double achromat, CVI Melles Griot). We kept the laser power at below 10 mW, and closed the shutter when not imaging, to minimize photobleaching. The combination of bright samples, low background, and efficient light collection produces images with high signal-to-noise ratio. Analysis of signal (image intensity of identified tubes) and noise (background near the tubes) was performed on the kymographs of Fig. S3,† yielding *μ*_signal_ = 510.4, *σ*_signal_ = 192.1, *μ*_noise_ = 37.4, *σ*_noise_ = 31.5, for a per-pixel signal-to-noise ratio of SNR = *μ*_signal_/*σ*_noise_ = 16.2 and a signal coefficient of variation of *c*_v_ = *σ*_signal_/*μ*_signal_ = 0.38.

#### Autofocus

The autofocus and temperature control features of our microscope were central in automating the data acquisition. A rotary motor (Z-drive, ASI) was mechanically coupled to the translation stage of the objective turret to control the vertical position of the objective *via* computer. We used an autofocus module in the μManager software^39,40^ to find and maintain the best-focus-position of the objective based on the image sharpness. A focused image has a higher sharpness than an out-of-focus image. The DNA nanotube images were sufficient for finding the best-focus position without the need for fiduciary beads. The autofocus method was robust for long time-lapse imaging. The μManager plug-in used the image sharpness function as feedback to the autofocus routine. We set the μManager to run the autofocus step either every 30 or 60 seconds to minimize photobleaching.

#### Temperature control

In our setup, we used two separate electronic circuits to control the temperature of the prism and the objective. Each setup was composed of a heating tape (Omega), a thermocouple (CHAL-005, Omega), and a temperature controller (Omega). We relied on the heat transfer from the heated objective and prism to achieve the desired temperature in the sample. This method produced highly reproducible sample temperatures. Consequently, in most experiments, we only measured the temperature of the prism and the objective. Two calibrated thermocouples (CHAL-005, Omega) were placed close to the field of view to calibrate the sample temperature as a function of both the prism and the objective. In this paper, we report the sample temperature based on our calibration table.

#### Flow chamber

A pre-cleaned glass capillary tube (Vitrotubes 5010, VitroCom) with inner dimensions of 100 μm × 1 mm × 5 cm was mounted on a 75 mm × 50 μm × 1 μm RCA-cleaned^41^ plain microscope slide (Corning, 2947-75 × 25) by applying a thin layer of 5 minute epoxy (Devcon) in between the two glass surfaces. The epoxy was left to cure at least overnight prior to imaging. The mounted capillary chambers were stored in ambient environmental conditions inside a microscope slide storage box and were used within a week. We believe that the small openings of the capillary chamber hinder contamination and consequently, it was safe to use the chamber as is, without any additional cleaning step.

We serendipitously discovered epoxy to be a convenient adhesive to mechanically and optically couple capillary tubes and microscope glass. First, the cured epoxy is inert with respect to immersion water; thus, the epoxy does not stain the water-immersion objective. Second, and more importantly, the refractive index of cured epoxy closely matches the refractive index of the microscope slide and the capillary tube. (Any refractive index mismatch increases the background signal due to more reflection.) An adhesive with a much higher refractive index than glass will shift the total internal reflection location to the interface between the adhesive and capillary glass surface. Conversely, an adhesive with a refractive index close to water will result in an evanescent wave at the boundary between the microscope slide surface and the adhesive. In the absence of adhesive, immersion water penetrates the cavity between the two glass surfaces, and the evanescent wave occurs at the microscope slide-immersion water interface instead of at the inner surface of the capillary tube where the fluorescent sample resides. Thus, cured epoxy between the capillary tube and microscope slide solves this problem.

### Polymerization mix

Our polymerization mix consists of pre-formed banded nuclei *i.e.*, two-color DNA nanotubes, supersaturated DNA tile solution, crowding agent, and buffer, as explained below.

#### DNA tile design

The DNA tile used here conforms to the “DAO-O” motif (double-crossover, antiparallel, odd–odd),^42^ which means that it is a double-crossover molecule. At crossover points, strands bend to become antiparallel to themselves. It has an odd number of DNA half-turns between crossover points in the same tile, and also an odd number of half-turns between neighboring tiles (middle). We used the sequence of a previously published DAO-E tile (double-crossover, antiparallel, odd-even; Fig. 1d, top left, of ref. 11) as the starting sequence for our DAO-O tile. In the new tile, we increased the distance between intermolecular crossover points by approximately half a turn of DNA, from 21 base pairs to 26 base pairs, which is equal to 5 half-turns. The new DNA tile has 2 pairs of 6-nucleotide sticky ends, instead of 5-nucleotide sticky ends, with the goal being to bring the nanotube formation temperature near 37 °C as required for enzymes used in a separate artificial cytoskeleton project.^9^ All of the original core and arm sequences were left unmodified during the sequence optimization. We used our custom MATLAB code to design the sequence for the extension of the arms and new pairs of sticky ends based on spurious binding minimization.^43^

#### DNA stock solution

Each DNA strand (synthesized by IDT DNA Technologies, Inc.) was resuspended separately and stored in purified water at a 10 μM stock concentration. To expedite the subsequent sample preparation step, we typically store our tile as an annealed DNA nanotube stock solution in a 4 °C refrigerator, and use it within 1 week after annealing. The stock of DNA nanotubes was made by mixing the four DNA strands at a final equimolar concentration of 1.5 μM each in a buffer consisting of 1× TAE [40 mM Tris–acetate and 1 mM EDTA (ethylenediaminetetraacetic acid)] with 12.5 mM Mg–acetate and then annealing from 90 °C to 20 °C at 1 °C min^–1^. In retrospect, we consider this annealing step to be unnecessary because of another annealing step in the preparation of the supersaturated DNA tile solution.

#### Pre-formed DNA nuclei with fiduciary markers

The simultaneous polymerization measurement of both DNA nanotube ends requires fiduciary markers. To create fiduciary markers, we pre-formed DNA nanotubes with random banding patterns to be used as nuclei. The banding pattern along the DNA nanotubes established fiduciary coordinates that enabled separate kinetic measurement of both ends of each DNA nanotube. The DNA nanotubes with fiduciary markers were prepared, as discussed below, from Cy3- and {Cy3, Cy5}-labeled nanotubes, which were called bright and dim bands, respectively. All of the tiles in the bright nanotubes were labeled with Cy3. For the separately prepared dim nanotubes, only 33% of the tiles were labeled with Cy3 and the remaining 67% were labeled with Cy5. Instead of using an unlabeled tile, we chose Cy5-labeled tiles to decrease the brightness of the tube fluorescence in the Cy3 channel. The aim was to minimize the physical difference between the Cy5-DNA and Cy3-DNA tiles so that both colors of tubes will be similar in terms of melting temperatures, kinetics, and other aspects relevant to the common core model of nanotube polymerization.

The DNA nanotube nuclei were prepared as follows: first, we annealed bright and dim DNA nanotubes separately at a tile concentration of 1.0 μM from 90 °C down to 50 °C at 1 °C min^–1^ and from 50 °C to 20 °C at 0.1 °C min^–1^. This annealing protocol produces DNA nanotubes with mean length on the order of 5 μm. On the same day, equal volumes of 1 μM bright and dim DNA nanotubes were fragmented into shorter nanotubes by subjecting the DNA nanotube mix to a high elongational fluid flow within a 20 μm × 20 μm constriction in a microfluidic chip^44^ at a 150 μL min^–1^ volumetric flow rate. The elongational flow near the constriction was sufficient to induce significant tension and induce DNA nanotube scission. The fragments had a mean size on the order of 1 μm. Subsequently, the stochastic end-to-end joining between fragmented bright and dim DNA nanotubes produced hybrid DNA nanotubes with random banding patterns^7,8^ that were later used as fiducial markers for the construction of kymographs.

**Fig. 3:**
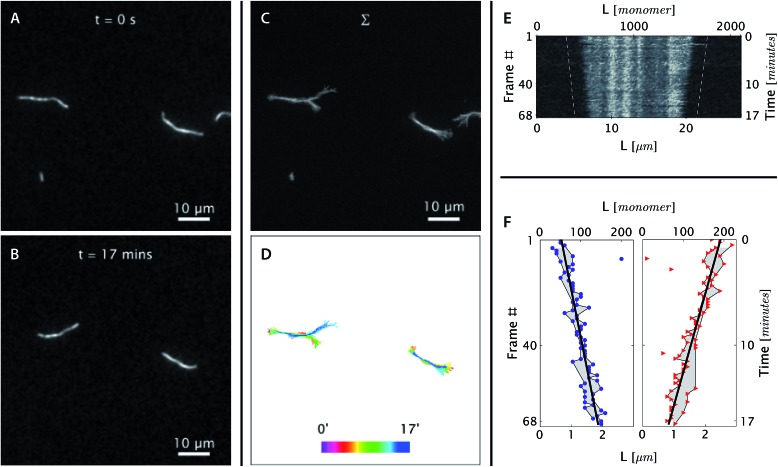
*Real-time observation of nanotube depolymerization*. (A and B) Before and after TIRF images of depolymerizing DNA nanotubes at 38.3 °C and 0 initial tile concentration. While the original images were at higher resolution, for display purposes they were intentionally blurred over a pixel radius of 4 here (1 pixel = 130 nm). Full resolution images appear in Movie S3.† (C and D) The superposition of the 69 images shows that the methylcellulose matrix confined the nanotubes close to the glass surface, where the evanescent illumination is maximum and focal plane is positioned. The nanotubes were still able to diffuse by reptation within the confinement space, mostly along the longitudinal axis of the nanotube and not sideways. During the period of observation, the tube on the left switched between two paths. The bottom middle panel shows the superposition of DNA nanotube traces at different time points. (E and F) The depolymerization is more noticeable when the curvilinear nanotube traces are straightened, aligned, and presented as a kymograph. Blue circles and red triangles indicate the outputs of the nanotube end detection algorithm. Isolated points indicate data that was rejected as outliers and not used in the linear fitting; the grey-shaded region identifies those points that were used for the fits. The linear fits of both straightened nanotube end positions are also presented as off-set white dashed lines in the top right kymograph. From the linear fit, the depolymerization rates for the left and right nanotube ends were measured to be 0.11 ± 0.01 and 0.14 ± 0.01 layers per second, respectively.

**Fig. 4:**
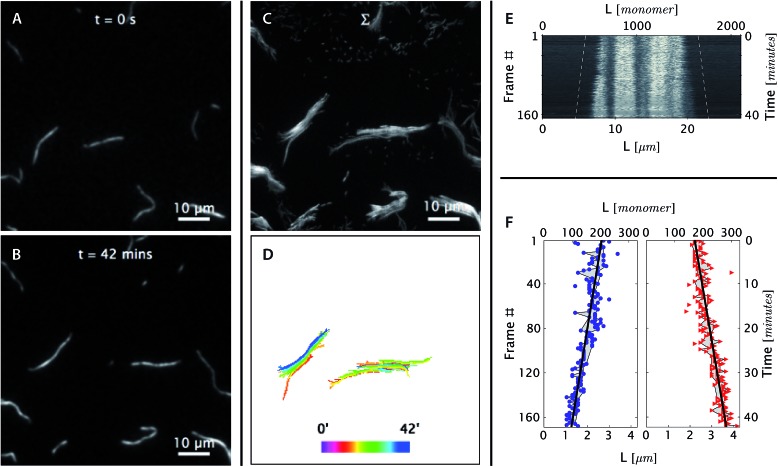
At [tile] > [tile]_crit_, DNA nanotubes grow at a constant polymerization rate. Panels are analogous to Fig. 3 except as stated. (A and B) Before and after images of DNA nanotubes after 42 minutes of polymerization at 38.3 °C and 400 nM initial tile concentration. The full image set is available as Movie S4.† (C and D) DNA nanotubes were mobile during the course of the experiment. The correlated displacement of all DNA nanotubes in the field of view could be due to the mechanical drift of our sample stage or due to settling of the methylcellulose matrix. The movement was very slow (<10 μm/40 min) and did not result in blurring of individual images. (E and F) The kymograph and linear fits of the right nanotube in the middle bottom panel support our expectation that at tile concentration above [tile]_crit_ at a given temperature, DNA nanotubes grow at a constant polymerization rate. From the linear fit, the growth rate for the left and right nanotube ends were determined to be 0.043 ± 0.003 and 0.040 ± 0.002 layers per second, respectively.

The bright and dim segments are visible in the microscopy images (the left panels of Fig. 3 and 4) and are more obvious in the kymograph (the right top panel of Fig. 3 and 4). As expected, the position of bright and dim segments did not move relative to each other during the course of data acquisition, which justified the choice of band positions along the DNA nanotubes to act as bonafide fiduciary markers.

#### Supersaturated DNA tile solution

For the polymerization assay, the supersaturated DNA tile solution was prepared by annealing 10 μL of Cy3-labeled DNA tile mix at 15/8× of the desired DNA tile concentration in 1× TAE/Mg^++^ from 90 °C to 50 °C. The slow annealing was halted at 50 °C in order to prevent spontaneous nucleation of nanotubes; for our experimental concentrations and time scales, DNA nanotube nucleation is not noticeable at temperatures above 40 °C. At 50 °C, 5 μL of 0.9% (w/v) methylcellulose (previously kept at 50 °C) was added to the 10 μL supersaturated DNA tiles. Note that the formation temperature depends on the tile concentration and on the annealing speed. By the end of the experiment, we determined that the observed formation temperature range is 35.2–38.3 °C, which corresponds to the temperature where spontaneous nucleation was observed in 100 nM and 500 nM samples after ∼5 minutes of imaging time, respectively. Thus, 50 °C incubation is above the formation temperature in any free monomer concentration used in this work. The sample temperature was then lowered to 45 °C. 2 μL of pre-formed banded nuclei were added at 45 °C and immediately the mix was gently injected into the capillary tube, which was already at the specified reaction temperature between the temperature-controlled prism and the objective. Both ends of the capillary chamber were immediately sealed with Vaseline to prevent evaporation.

#### Crowding agent confines the nanotubes close to the glass surface

We included 0.3% (w/v) methylcellulose (viscosity 4000 cP at 2% in H_2_O at 20 °C, purchased from Sigma-Aldrich M0512) as a crowding agent to confine DNA nanotubes near the bottom (as well as top and side) of the glass surface where the focal plane and evanescence field were positioned. In a crowded environment, the entropy of the system is maximized when all of the long structures are pushed close to another surface, such as capillary tube walls. This entropic confinement did not hinder the mobility of confined DNA nanotubes within their confinement space (middle columns of Fig. 3 and 4). This behavior is in accord with previous observations of confined biopolymers in the presence of crowding agents.^45,46^ The side effect of this confinement strategy is that the same entropic force also favors confining DNA nanotubes to other surfaces, including the surfaces of other DNA nanotubes. Consequently, at high DNA nanotube densities, DNA nanotubes were observed to exhibit side-to-side joining (Movies S1 and S2†) and lateral aggregation. The increasing intensity of tubes in images corresponds to the lateral “bundling” of multiple DNA nanotubes or side-to-side joining (observed directly in Movies S1 and S2†).

### Data acquisition

Since polymerization is temperature sensitive, we paid close attention to the temperature of our sample and minimized exposure to the room temperature.

Before the injection of DNA monomers, the empty sample chamber was mounted onto the heated prism and under the heated objective and immersion water to bring the sample chamber to the desired steady state temperature. Skipping this step will result in a sample chamber that is initially at room temperature, which would cause DNA nanotubes to nucleate very rapidly. In addition, our autofocus did not work well when the temperature of the sample, prism, and objective changed rapidly, such as in the initial heating step of our method of temperature control. The chamber was left empty at the desired steady state reaction temperature until the polymerization mix was ready.

In contrast to adding a liquid sample to a filled chamber, injecting a sample into an empty capillary chamber results in a known initial sample concentration. Previously, studies that used a similar sample chamber would flush the filled chamber with at least twice the chamber volume to ensure that the reaction conditions held during measurements. Because the fluid flow approaches zero near the channel walls, it is difficult to produce samples with known concentration using that method. The second advantage of starting with an empty chamber is fast injection time. Due to stronger capillary action, injecting the sample into an empty chamber requires less time than infusing a filled chamber with sample. The fast injection may also be important in minimizing thermal contact between the heated liquid of DNA tiles, DNA nanotubes, and the ambient room temperature. However, an empty chamber also possesses an intrinsic problem; the fast injection flow of DNA nanotubes, especially at high temperatures, induces DNA nanotube scission. We minimized the scission problem by adding the sample gently at the opening of the empty chamber. The injection time was approximately 5 s for ⪅6 μL sample. Quantifying how much scission occurs with our injection protocol is not necessary since the polymerization rate measurements should be independent of the initial amount of fragmentation.

We identified three instances in our protocol in which the polymerization mix was exposed to ambient environment. First, we pipetted 5 μL methylcellulose to a supersaturated tile solution with a pipette tip that was at room temperature. Second, after we incubated the solution of supersaturated tiles and methylcellulose at 50 °C, we took the sample out from the temperature cycler and mixed it for ≈5 s at room temperature. Third, we injected the supersaturated tiles, methylcellulose, and pre-formed DNA nuclei at the opening of the heated glass capillary chamber with a pipette tip that was not heated. To minimize potential problems, such as the rapid nucleation of DNA nanotubes from supersaturated DNA tiles before the sample was injected to the heated glass capillary chamber, we performed these three steps as rapidly as we could. The typical execution time for these steps was 5 s and no longer than 10 s. In almost all cases, the fast sample handling seemed to be sufficient to avoid spontaneous nucleation before imaging, with the exception of a polymerization assay at 600 nM and 41.4 °C. We therefore did not use data taken at 600 nM.

#### DNA nanotube imaging

Our prism-based TIRF microscope, equipped with temperature control and automated focusing, monitored the dynamics of the DNA nanotubes that were confined close to the glass surface (Fig. 3 and 4) by imaging with a 100 ms exposure time and 2–4 frame per min acquisition rate. The signal-to-noise ratio was very high, even in the presence of a high concentration of Cy3-labeled free monomers in solution. For all of the nanotubes that were analyzed, we did not encounter any pausing of polymerization in any of our movies, which provides evidence that the untreated glass surface is not too sticky. The majority of DNA tiles were in the free monomer state. The typical total concentration of DNA tiles in pre-formed DNA nuclei was approximately 7 nM, which is more than 10× smaller than the most dilute free monomer concentration in our assay (100 nM). Even after 2 hours of imaging, we typically observed a difference of less than a factor of 2 in contour length for all DNA nanotubes, which corresponded to a small DNA tile concentration change.

Under reaction conditions where spontaneous nucleation was hardly observable, DNA nanotube polymerization was followed for at least 1 hour and no longer than 2 hours. Much to our surprise, our imaging protocol did not require an oxygen scavenger buffer to achieve and maintain a high signal-to-noise ratio for the full duration of time-lapse imaging. At an acquisition rate of typically 4 frames per min, we usually acquired enough data points in less than 30 minutes. If significant spontaneous nucleation was observed, we terminated the data acquisition after ∼5 minutes because the newly formed nuclei rapidly obscured the visibility of the pre-formed nuclei. Moreover, the new nuclei can also end-to-end join to a growing DNA nanotube end, which would have made our polymerization rate measurements unreliable. Thus, spontaneous nucleation limited the range of temperatures and concentrations for which we could obtain accurate rate measurements. The range of hysteresis observed in UV absorbance annealing and melting curves at 200 nM (Fig. S1†) predict a similar range of temperatures for which the DNA nanotubes can be held out of equilibrium during the time required for a typical movie.

### Data analysis

The polymerization rate discussed here refers to the elongation or shortening of DNA nanotubes (and thus is measured in layers per second) as opposed to the rates of association and dissociation of a single DNA tile to a single binding site. (The polymerization rate constant *k*_on_ is roughly half the association rate constant for a single site, *k*siteon, under usual growth conditions where there are on average *m*/2 available binding sites in a nanotube that is *m* tiles in circumference. For the same reason, the depolymerization rate constant *k*_off_ is roughly half the dissociation rate constant for a single site, *k*siteoff,2. This distinction is discussed more in the Discussion section and in the ESI analysis of Hypothesis 2, pages S8–S12.†) A layer is defined as a ring of *m* tiles for a *m*-tile-wide nanotube; it is estimated to be the length of 42 bp's or 14 nm (accounting for the expected curvature of the helix axis^47^). In our analysis, the number of layers in a nanotube is calculated by dividing its length by 14 nm. The polymerization rate was measured using two methods: (1) kymographs^48^ or (2) length measurements taken at two frames with a sufficient time difference. The kymograph allows separate measurement of both nanotube ends at the cost of time to construct a kymograph. Obtaining the polymerization rate from the nanotube length at two data points is fast but can only measure the net polymerization rate of a nanotube end. Although alignment of fiducial markers is reliable in multi-frame kymographs, image shot noise precludes reliable alignment in two-frame measurements and any asymmetry in the polymerization rate at the nanotube ends will be unobservable.

In the first method, we applied an ImageJ^40^ plugin developed by Kuhn and Pollard^48^ to construct kymographs from a series of DNA nanotube images. We used their image analysis routine to convert a rough hand trace of each DNA nanotube to a refined trace of the nanotube by snapping each pixel along the trace to the DNA nanotube axis. The intensity along the refined traces was used to construct equivalent straightened images of the curvilinear DNA nanotubes. The straightened images of the same nanotube at different time points were aligned and stacked into a kymograph. We wrote Mathematica (Wolfram Research) code that shifted the longitudinal offset between straightened DNA nanotubes until the sum of the correlations between straightened images in a kymograph was maximized, *i.e.*, the banding patterns were vertically aligned. The longitudinal position of both nanotube ends in a kymograph was detected by setting a chosen threshold for both DNA nanotube ends, typically less than the half maximum value of any given straightened images. For each nanotube end, we performed a linear fit through the coordinates of nanotube ends in the aligned image to measure the polymerization rate (Fig. 3, 4, and S3†). The curve fitting was computing in Mathematica using the LinearModelFit function. Outliers were detected by calculating the Euclidean distance between the measurement and the estimates. For quality control, data points that are >5 pixels (649 nm) away from the estimates were excluded from the curve fitting. Error bars represent standard errors (1*σ*).

In the second technique, we simply calculated the net polymerization rate from the ratio of the length change between two frames and the time interval between the frames. Because the kymograph integrates over multiple frames, its standard error is likely to be smaller. However, the simpler technique is far quicker than constructing a kymograph for each DNA nanotube, and, by bypassing the alignment process, we could measure the rates from DNA nanotubes that did not have multiple bands.

The accuracy of our measurements was not limited by the per-pixel signal-to-noise of microscopy imaging (*μ*_signal_/*σ*_noise_ = 16.2). Rather, variability of pixel brightness within the straightened nanotube images (*c*_v_ = *σ*_signal_/*μ*_signal_ = 0.38, which is presumably attributable to shot noise, fluorophore blinking, and processing to straighten nanotube images) limited the accuracy of locating nanotube ends and aligning frames in kymographs. Presuming that each nanotube end is localized to within 1 or at most 2 pixels, we expect the length measurements to have an error of less than 0.52 μm (given pixel size 130 nm). The empirical localization errors (per-frame root-mean-square residuals from the linear fits in the kymographs of Fig. 3F, 4F, and S3†) were comparable (*σ*_L_ = 0.34 μm).

#### Statistical analysis

**Fig. 5:**
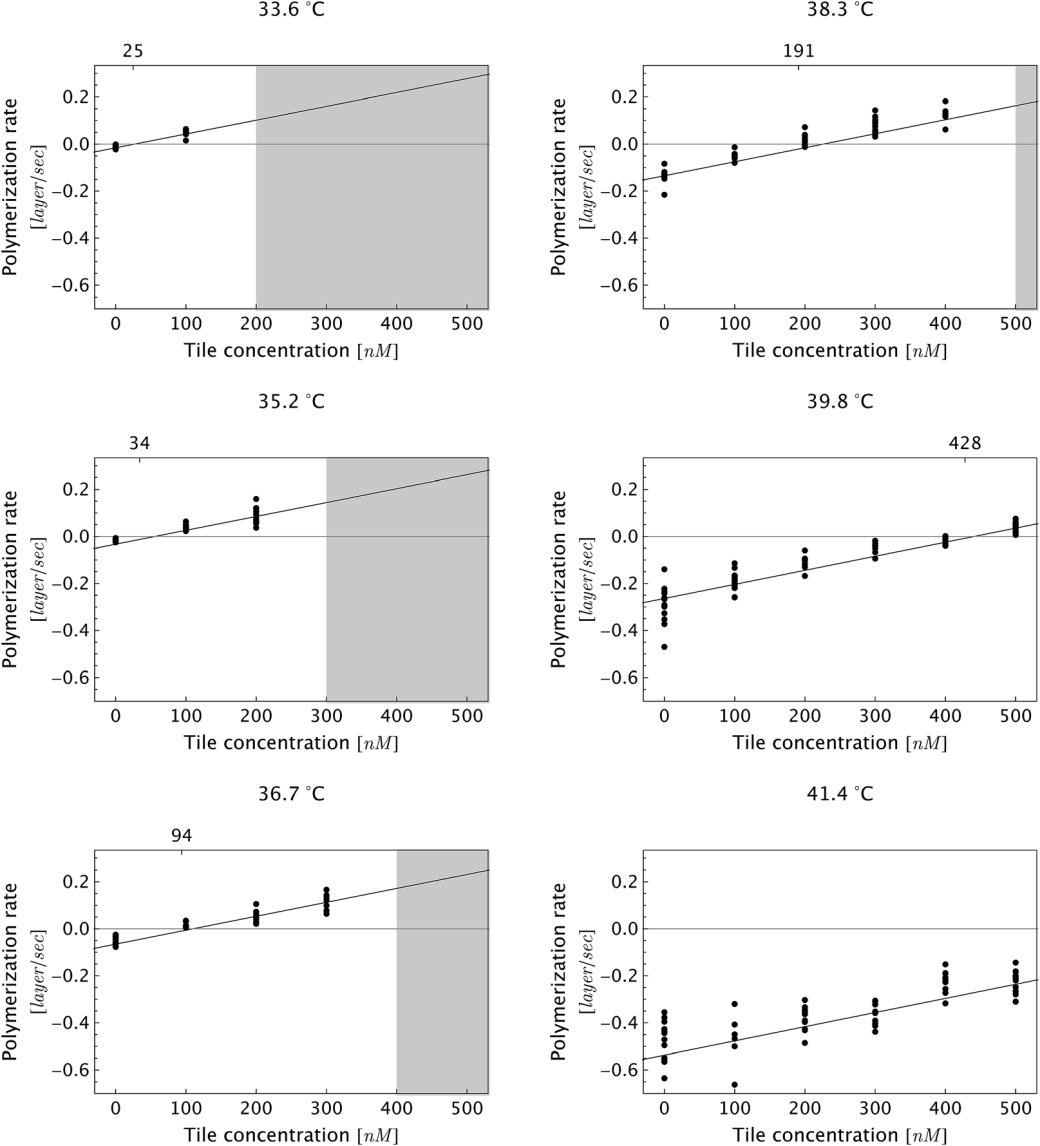
Dependence of DNA nanotube polymerization rates on free tile concentration for several reaction temperatures. As expected, the polymerization rate was faster at lower temperatures and higher free monomer concentrations. The gray-shaded region represents the parameter space where we observed spontaneous nucleation and end-to-end joining, which invalidates measurements due to side-to-side joining between pre-formed nuclei and the newly nucleated nanotubes. Furthermore, the side-to-side joining obscured the time evolution of individual DNA nanotubes. As a consequence, the movies in the shaded parameter space were not analyzed. The fitting line is the global linear fit (eqn (1)). The numbers on the top horizontal axis are the inferred critical monomer concentrations (in nM), which were calculated by setting eqn (1) to zero at given temperature *T*. The data at a given temperature and at different monomer concentrations was fitted separately (not shown), and the fitting results are presented in Table S2.†

**Fig. 6:**
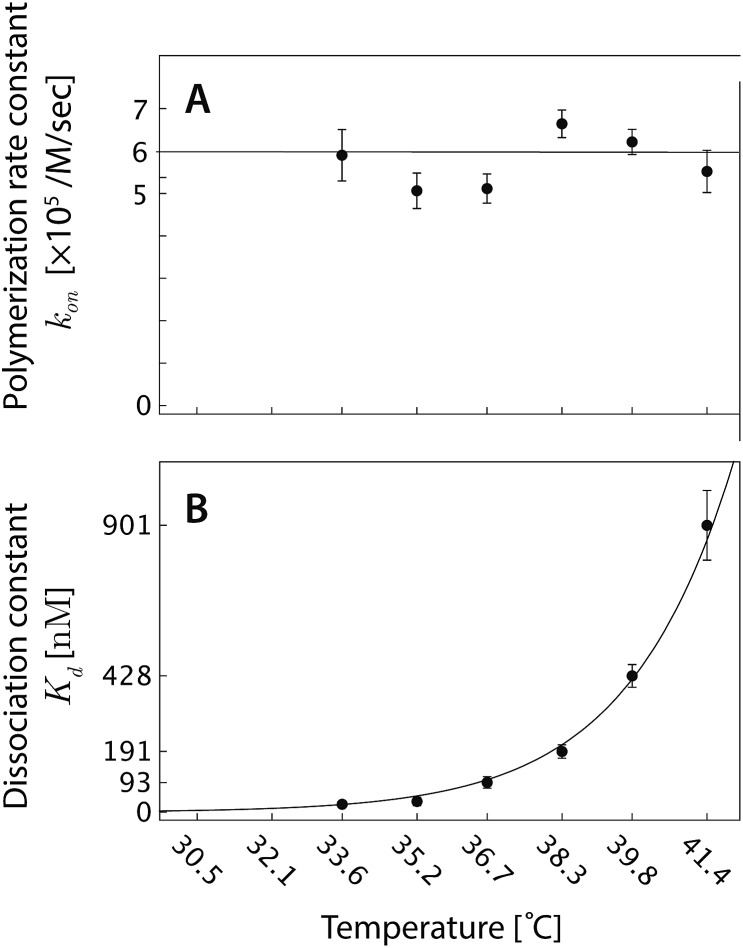
(A) The polymerization rate constant, *k*_on_, as inferred from independent “local” linear fits at each temperature, is relatively constant. (B) The inferred equilibrium dissociation constant, *K*_d_, for DNA nanotube polymerization grows exponentially with the temperature. The dissociation constant was calculated by taking the ratio of inferred *k*_off_ and *k*_on_ parameters from the local fits. The solid line was computed by employing the Δ*H*° and Δ*S*° parameters from the global data fit and eqn (2).

Curve fitting was computed in Mathematica (Wolfram Research) using the command NonlinearModelFit. For global fits, we subjected all of our data (*N* = 347 nanotubes) to eqn (1) and (2) for plots shown in Fig. 5, 6 and S2.† For local fits (Fig. 6 and Tables S1 and S2†), each curve fitting was limited to the data obtained at the indicated monomer concentration (Table S1†) or temperature (Table S2†). In the Δ*G*°37 analysis in the discussion section, the Δ*H*° term in eqn (2) was substituted by Δ*H*° = Δ*G*°37 – (273.15 + 37 K) × Δ*S*°. In all curve fitting, all data points were weighted equally. The fitting routine minimizes the sum of the squared differences between the model's predicted rate and the experimentally-measured rate. The results from the global and local fits are reported as mean ± standard error (1*σ*) for each parameter estimate.

## Results

### Polymerization rate measurements

At the critical monomer concentration [tile]_crit_, the tile attachment rate and the tile detachment rate are equal. As a result, the length of DNA nanotube *i* fluctuates around its length *L*_*i*_. At a constant tile concentration away from [tile]_crit_, DNA nanotubes either elongate or shrink at a constant rate. The resolution of the microscopy assay was diffraction limited at ∼250 nm or ∼18× the length of a DNA tile (∼14 nm; Fig. 1). Our imaging optics produced movies that were sufficient to accurately track both ends of individual DNA nanotubes. However, the optics were insufficient to discriminate the precise tile arrangement at nanotube ends and could not detect individual tile attachment and detachment events.

The polymerization rate was measured from time-lapse images (Fig. 3 and 4) by (1) constructing kymographs and (2) measuring DNA nanotube lengths at two time points. We address the results and merits of both approaches below.

#### DNA nanotubes depolymerize at a steady rate at low free tile concentration ([tile] < [tile]_crit_)

To measure the rate at which monomers dissociated from DNA nanotubes *k*_off_, we diluted 1 μM of DNA tiles (as pre-formed DNA nanotubes) at room temperature by a dilution factor of 143 in imaging buffer [1× TAE/Mg^++^, 0.3% (w/v) methylcellulose]. In the depolymerization experiments with non-zero free DNA tile concentrations, we added Cy3-labeled DNA tiles to the pre-formed DNA nanotube nuclei.

Fig. 3 shows the steady-state shortening of DNA nanotubes at 38.3 °C at [tile] = 0. Since the critical monomer concentration is always positive, DNA nanotubes are expected to depolymerize at [tile] = 0. The free tile concentration will increase as depolymerization increases, but this is limited by the total concentration of monomers within the pre-formed DNA nanotube nuclei, which was just 7 nM. As this limit won't be reached unless all nanotubes completely depolymerize, and as the concentration interval in our experiments was substantially larger at 100 nM, we considered deviations from the nominal free tile concentration to be negligible.

#### DNA nanotubes elongate at high free tile concentration ([tile] > [tile]_crit_)

To measure the second-order forward rate constant *k*_on_ with which DNA tiles associated to DNA nanotube ends, we assayed the DNA nanotube polymerization at multiple DNA tile concentrations with intervals of 100 nM and at multiple temperatures ranging from 28.9 to 41.3 °C. An example of DNA nanotube elongation is shown in Fig. 4 for the case of a tile concentration of 400 nM and a temperature of 38.3 °C. In these experiments, the net elongation results from the excess of tile association events over tile dissociation events.

As in the depolymerization case, changes in the free tile concentration during the experiment can be considered negligible. Since reaction conditions were chosen such that nanotubes did not double in length during the experiment, and such that spontaneous nucleation was very rare, the decrease in free tile concentration can again be bounded by the total tile concentration within the pre-formed nuclei, *i.e.*, 7 nM, which is less than 10% of the smallest non-zero concentration in our experiments.

#### DNA nanotubes polymerize at steady rates

The linear fits of DNA nanotube end positions in Fig. 3 and 4 show that both polymerization and depolymerization of DNA nanotubes proceeded at steady rates. Surprisingly, the kymographs of DNA nanotube polymerization reveal the relatively high prevalence of apparently asymmetric polymerization rates between the two ends of an individual DNA nanotube. This phenomenon, and its statistical significance, is considered further in the Discussion.

### Kinetic and thermodynamic analysis of DNA nanotube polymerization

To test the common core nanotube polymerization model, we measured the polymerization rates of 347 DNA nanotubes within a 0–500 nM concentration range and a 28.9–41.3 °C temperature range. Having established confidence in the steady polymerization rate, average polymerization rates were measured by comparing the nanotube lengths at two time points determined to be sufficiently far apart (Δ*t* > 10 min). We excluded DNA nanotubes that had undergone spontaneous scission, end-to-end joining, or side-to-side joining from our data set (observed directly in Movies S1 and S2†).

The dependence of polymerization rates 
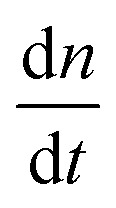
 on free monomer concentrations [tile] at different temperatures *T* (summarized in Fig. 5 and S2†) was compared to the ‘common core’ model, where1
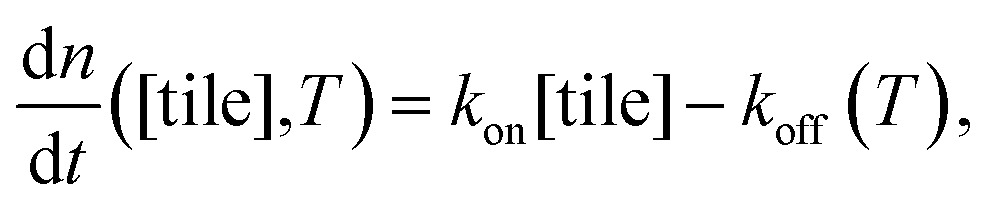
with

2 *k*_off_(*T*) = *k*_on_ e^–(Δ*H*°–*T*Δ*S*°)/*RT*^ × *u*_0_

and *n* is the number of tile layers in a DNA nanotube, *t* is time, *k*_on_ is the polymerization rate constant, *k*_off_ is the depolymerization rate constant, *T* is the temperature in Kelvin, Δ*H*° is the standard enthalpy of tile-DNA nanotube dissociation, Δ*S*° is the standard entropic cost of tile – DNA nanotube dissociation, and *u*_0_ = 1 M is the standard concentration assumed for the thermodynamic parameters. In this model, we ignored the plausible kinetic and thermodynamic parameter differences between DNA nanotubes of different circumferences; in the Discussion they are considered in the context of asymmetric growth rates.

From a single global fit to all the data in Fig. 5, the polymerization rate constant *k*_on_, was inferred to be (5.99 ± 0.15) × 10^5^ M^–1^ s^–1^. This nonlinear fit also gave the thermodynamic parameters of the combined polymerization data to be Δ*H*° = 87.9 ± 2.0 kcal mol^–1^ and Δ*S*° = 0.252 ± 0.006 kcal mol^–1^ K^–1^. The free energy near the experimental temperatures was more accurately determined than the enthalpy: repeating the global nonlinear with free variables *k*_on_, Δ*S*°, and Δ*G*°37 yielded Δ*G*°37 = 9.84 ± 0.02 kcal mol^–1^.

As a comparison to the global fitting results, the polymerization rates at a given temperature *T* were subjected to linear fitting to obtain 
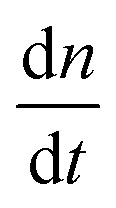
 as a function of tile concentration:3
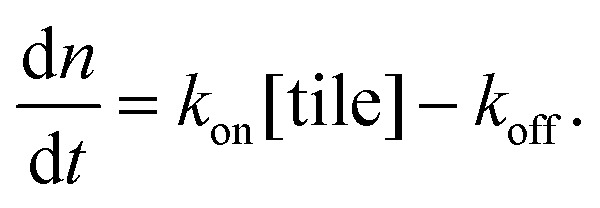
Where independent “local” values of *k*_on_ and *k*_off_ were fit for each temperature, and eqn (2) was not used. The inferred polymerization rate constant, *k*_on_, and equilibrium dissociation constant, 
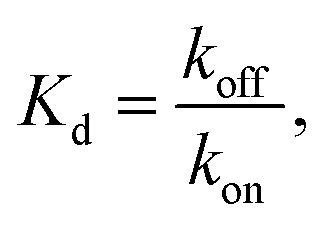
 from the local linear fittings were plotted against the reaction temperature in Fig. 6 and summarized in Table S2.† The inferred values from local fits (solid circles) are in agreement with the theoretical model (solid line) using the globally inferred values of *k*_on_, Δ*H*°, and Δ*S*°.

## Discussion

To summarize our measurements, we developed a TIRF microscopy assay to directly observe the polymerization dynamics of single DNA nanotubes for up to 2 hours of imaging over a wide range of DNA tile monomer concentrations and temperatures. The long duration of time-lapse imaging required stable temperature control and an autofocusing system. The polymerization rates were measured by two methods: (1) constructing a kymograph from straightened traces of DNA nanotubes and (2) measuring the nanotube length change between two time points. The first method was able to simultaneously obtain the polymerization rates for both ends of the filaments and confirmed that each end depolymerizes (Fig. 3) or polymerizes (Fig. 4) at a steady rate. The second measurement strategy was used to analyze a much larger number of DNA nanotube polymerizations (*N* = 347) for extracting kinetic and thermodynamic parameters *via* global fitting.

### Interpretation of the measured polymerization and depolymerization rate constants

The common core model for the polymerization of DNA nanotubes was compared to the kinetic Tile Assembly Model (kTAM) developed for theoretical study and simulation of algorithmic DNA tile self-assembly.^49^ Whereas the common core model uses the polymerization rate constant *k*_on_ to model the concentration dependence of the net nanotube elongation rate (in layers per second) and the depolymerization rate constant *k*_off_ to model the net rate of shrinking (also in layers per second), the kTAM models the growth and shrinking process at the level of individual tile addition and removal steps. In the kTAM, the association between a free tile and a binding site is assumed to be a reaction with forward rate

**Fig. 7:**
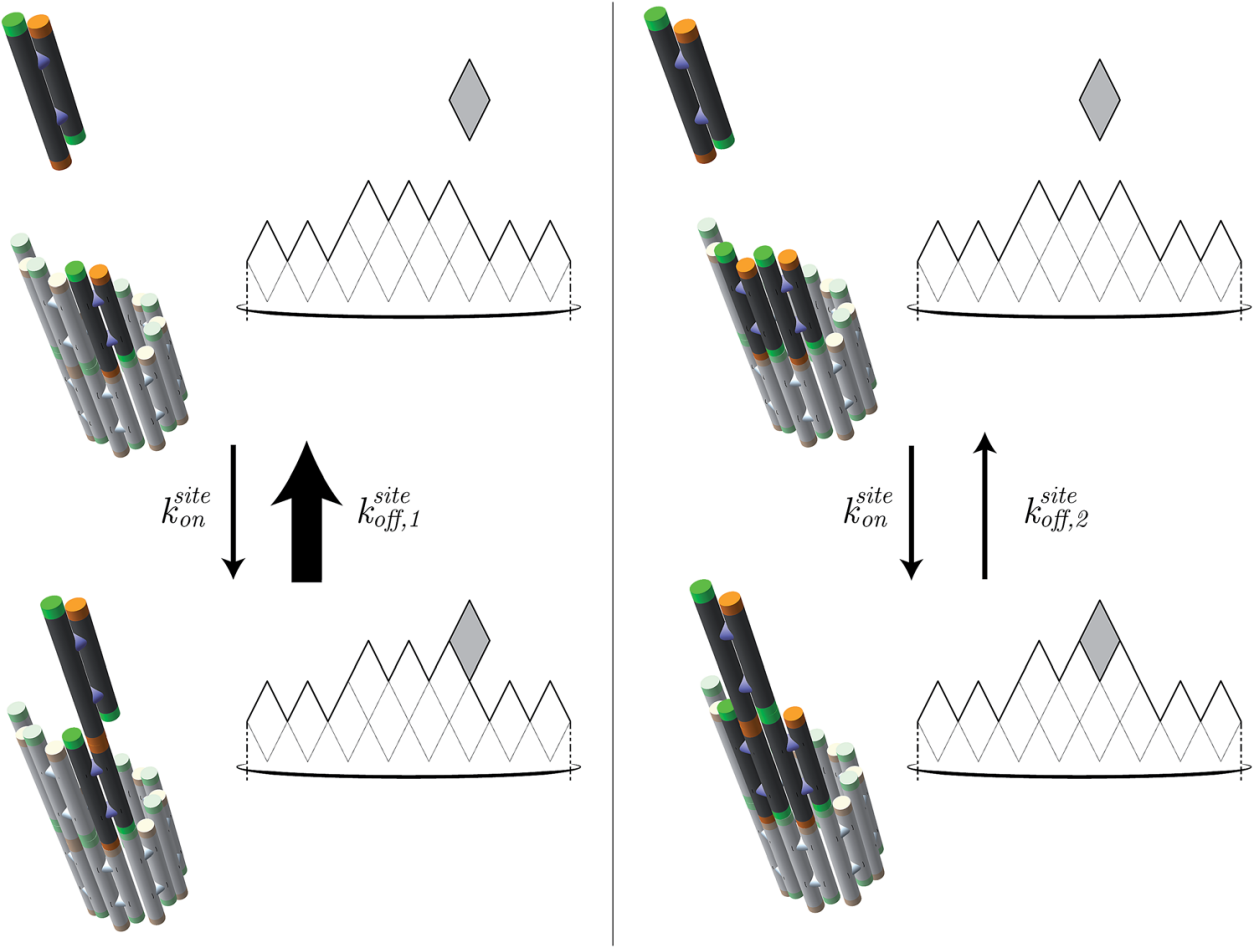
In the kinetic Tile Assembly Model,^49^ the rate of a free tile attachment to an available site in DNA nanotube end *k*siteon is independent of the number of sticky ends in the potential binding site. To satisfy detailed balance, the reverse rates depend on the number of available sticky ends. Hence, a DNA tile that only binds with one sticky end (left panel) will dissociate from a DNA nanotube faster than DNA tile with 2 bonds (right panel). The configuration of DNA nanotube ends and the position of dark tiles are different between left and right panels. Here, the highlighted attachment sites in the left and right panels provide 1 and 2 sticky ends, respectively. The attachment sites are illustrated as darker colored tiles. The sticky ends, illustrated as short green or orange tubes, are complementary when the colors match. The faster rate is indicated by the larger arrow of *k*siteoff,1 than *k*siteoff,2.

4 *r*_f_ = *k*siteon[tile],

where *k*siteon is the second-order association rate constant for an individual tile to an individual site. The reverse reaction rate depends on the stability of the binding and is modeled to be5
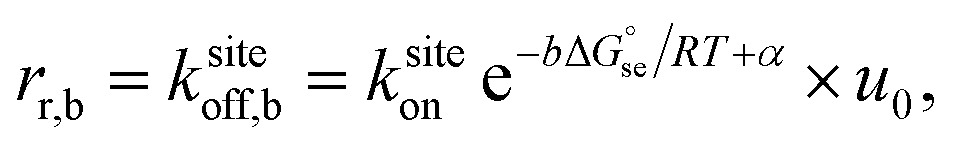
where *b* is the number of sticky end bonds, Δ*G*°se > 0 is the standard free energy for breaking a single sticky end bond at standard concentration *u*_0_ = 1 M, and *αRT* is the initiation energy for double-stranded DNA formation with *α* ∼ ln(20) in solution.^49^ The standard free energy Δ*G*°se can be further expressed as Δ*G*°se = Δ*H*°se – *T*Δ*S*°se. In the kTAM, due to the weak bond strength of one sticky end interaction, a tile that binds with one sticky end will quickly disassociate from the nanotube end, as illustrated by the large arrow in the left panel of Fig. 7. In DNA nanotube polymerization, configurations where an incoming tile can bind with 3 or 4 bonds can be neglected because a DNA nanotube end is highly unlikely to contain any tile arrangement allowing for a tile to bind with 3 or 4 sticky ends. For the quantitative analysis, we ignored 1, 3, and 4 sticky end interactions and assigned the inferred Δ*G*° as the free energy of an interaction with two sticky-ends Δ*G*° = 2Δ*G*°se – *αRT*, Δ*G*° > 0 (Fig. 7 right).

Under the assumption that tiles attach or detach only when bound by exactly 2 sticky ends, we derive (ESI, pages S8–S12†) that steady-state growth (or shrinkage) results in on average *m*/2 potential attachment sites and on average *m*/2 potential detachment sites, for nanotubes of large enough circumference *m*. Since *m* tiles must attach (or detach) to grow (or shrink) by one layer, we have *k*_on_ = *k*siteon/2 and *k*_off_ = *k*siteoff,2/2. Thus the thermodynamic and kinetic parameters of the kTAM can be put in exact correspondence with those of the common core model.

### Symmetrical or asymmetrical polymerization?

**Fig. 8:**
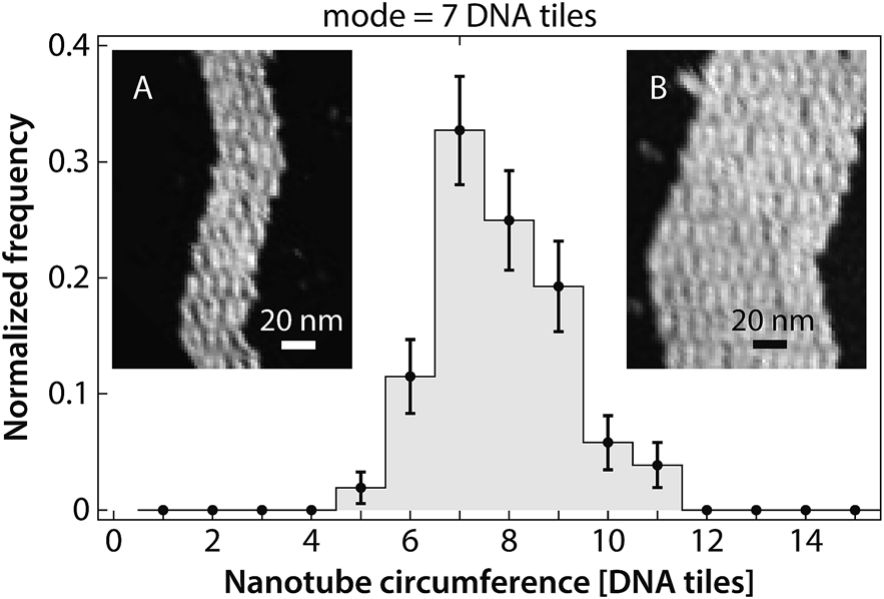
Our annealing protocol produces DNA nanotube nuclei that are 5–11 tiles in circumference, with 7-tile-wide DNA nanotubes as the most prevalent. Images of individual opened DNA nanotubes for constructing this histogram are presented in the (Fig. S4†). Heterogeneous circumferences were also observed in the *in vitro* self-assembly of other tubular structures, such as protein microtubule.^50–52^ The error bars are the standard deviation for each bin calculated using a bootstrapping method. (Insets) Representative images of opened DNA nanotubes with circumference of 5 (A) and 11 (B) DNA tiles.

Many kymographs exhibited asymmetrical growth rates in the two ends (Fig. S3†). Asymmetrical growth could be expected for several reasons. For example, one end of the nanotube presents sticky ends 1 and 2*, while the other end presents sticky ends 1* (which is fluorophore-labeled) and 2. Thus, although the thermodynamics of adding tiles must be the same for both ends, the kinetics could be different. Alternatively, even if constant-diameter nanotubes have identical kinetics on both ends, growth rates could differ in nanotubes that were formed by joining events^8^ between precursor nanotubes of two distinct diameters. Under our annealing protocol for creating nuclei, nanotube diameters varied between 5 and 11 tiles in circumference (Fig. 8 and S4†). Both kinetic factors related to tile assembly pathways, as well as thermodynamic factors related to nanotube strain,^7^ could differ for tubes of different circumferences, although the latter factors are likely to be more significant (Fig. S5 and S6†). However, statistical analysis suggests that some (or all) of the experimentally observed asymmetry can be attributed to noise in the rate measurements (Fig. S7†), and thus none of these potential effects are likely to factor largely in our experiments. Using DNA origami seeds^22,23^ that allow growth from only one side, rather than pre-formed nanotube nuclei that can grow on both ends, would in principle allow this issue to be resolved.

### Comparison with previously reported kinetic and thermodynamic parameters

#### Kinetic parameters

We measured *k*_on_ to be (5.99 ± 0.15) × 10^5^ M^–1^ s^–1^ for the polymerization model. As noted earlier, not all parts of growing/shrinking nanotubes contribute to the polymerization/depolymerization. Only sites with 2 sticky ends contribute to the process (Fig. 7 and S5–S6†). Consequently, the inferred association rate constant for a single DNA tile binding to an available site at the end of a DNA nanotube was expected to be *k*siteon ≈ 2 × *k*_on_ ≈ 1.2 × 10^6^ M^–1^ s^–1^. As an oversimplification, we initially compare the association rate constant for a tile attaching to a site by two length-six sticky ends, *k*siteon, to the second order association rate *k*_2_ for a 12 nucleotide DNA strand hybridizing to its perfect complement. Wetmur and Davidson^53,54^ determined 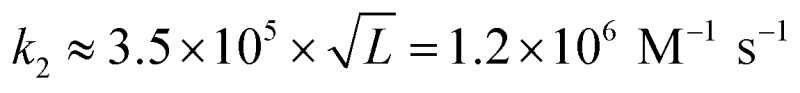 for *L* = 12; Morrison and Stols measure *k*_2_ ≈ 10^7^ M^–1^ s^–1^ for a 10-mer;^55^ while Zhang and Winfree obtained the range of forward rate to be (1–6) × 10^6^ M^–1^ s^–1^ for toeholds of various sequences and lengths up to 15 nucleotides.^56^ Thus, to first approximation, tile association rate constants appear to be comparable with oligonucleotide hybridization rate constants.

Our results may also be compared to previous investigations of the kinetics of tile assembly. In two separate works with different types of DNA ribbon, Schulman and Winfree^13^ as well as Fujibayashi and Murata^28^ used the values suggested in the original kTAM paper^49^ (or within 25% thereof, *i.e.* 0.8–1.0 × 10^6^ M^–1^ s^–1^) for the tile association rate constant in their kTAM-variant simulations, yielding satisfactory (though indirect) agreement with experimental data. In contrast, Chen *et al.*'s study of “snaked tile” growth and facet nucleation^26^ increased *k*siteon to 17 × 10^6^ M^–1^ s^–1^ in order to match their experimental data; this is 14× faster than our measurement. However, given the sensitivity of their complex simulation to both *k*siteon and to the various sticky-end free energies, none of which were determined experimentally, we presume that their inferred value for *k*siteon is unreliable. The final comparison is to Jiang *et al.*'s careful study of the kinetics of dimer association for a variety of DX and DX-like tiles.^57,58^ This careful study found that at 24 °C, dimers binding by two 5 nt sticky ends formed at approximately twice the rate as dimers binding by a single 10 nt sticky end (roughly 2.5 × 10^6^ M^–1^ s^–1^ *vs.* 1.2 × 10^6^ M^–1^ s^–1^). They also studied the temperature dependence of these rates, down to 12 °C, from which we extrapolate a rate of 3.5 × 10^6^ M^–1^ s^–1^ for association by two sticky ends at 37 °C, which is within the range of temperatures in our study. The roughly three-fold difference, compared to our results, could be attributed to sequence dependence or induced strain due to the DNA nanotube context, as discussed in the thermodynamics section below.

#### Thermodynamic parameters

To assess the experimentally-measured thermodynamic parameters in our study (Table S1†), we compared the enthalpy, the entropy, and the standard free energy of two sticky end interactions at 37 °C, to the theoretical predictions and to values from previously reported studies of the free energy of DNA hybridization. The first comparison that we examine is with respect to the kTAM.^49^ In that model, the enthalpy of disassembly was estimated to be Δ*H*° = *R* × *sb* × (4000 K), where *s* is the number of base pairs in a sticky end and *b* is the number of sticky end bonds. For *b* = 2 and *s* = 6, the simple expression yields the value of Δ*H*° = 95 kcal mol^–1^, which is within 10% of our measurement of Δ*H*° = 87.9 ± 2.0 kcal mol^–1^. For the entropy, using the kTAM and *α* = ln(20),^49^ the theoretical value was predicted to be Δ*S*° = *R* × (11*sb* + *α*) = 0.268 kcal mol^–1^ K^–1^, which is within 10% of the measured Δ*S*° = 0.252 ± 0.006 kcal mol^–1^ K^–1^ in our experiment. A more insightful thermodynamic parameter to describe the polymerization process is the standard free energy. Using the predicted and experimentally-measured values of Δ*H*° and Δ*S*°, we calculated the standard free energy of two sticky end interactions at 37 °C to be Δ*G*°37 = Δ*H*° – [(273 + 37) K]Δ*S*° = 12.2 kcal mol^–1^. In our experiment, the global data fit yielded the free energy Δ*G*°37 = 9.84 ± 0.02 kcal mol^–1^. The Δ*G*°37 from the kTAM is 3.8*RT* higher than our measured Δ*G*°37.

To our knowledge, the only published values for thermodynamic parameters of double crossover tile-based DNA crystal structures in solution were obtained from bulk studies of DNA ribbons of designed widths.^13^ The ribbons were composed of multiple tiles with 5 bp sticky ends, which is shorter than the 6 bp sticky ends in our tiles. Schulman and Winfree extracted Δ*H*° and Δ*S*° from a series of UV absorbance data by van't Hoff analysis. They measured Δ*H*° = 102.4 kcal mol^–1^ and Δ*S*° = 0.300 kcal mol^–1^ K^–1^. To compare our measurements to these values, we multiplied these values by 6/5, which is the ratio of sticky end lengths of our DNA nanotube and Schulman and Winfree's ribbon. The linear extrapolation gives Δ*H*° = 122.9 kcal mol^–1^ and Δ*S*° = 0.360 kcal mol^–1^ K^–1^. Using these adjusted Δ*H*° and Δ*S*° values, the free energy was calculated to be Δ*G*°37 = 11.3 kcal mol^–1^, which is 2.4*RT* higher than our measured Δ*G*°37.

One class of thermodynamics measurements relevant to DNA nanotube polymerization is the dimerization reactions of rigid DNA molecules, such as (1) quadruple-crossover (QX) molecules^59^ and (2) double-crossover (DX) DNA tiles.^60^ The QX molecule, in essence, is a sheet of 4 parallel DNA helices. By attaching different 6 bp sticky ends to a QX pair, the thermodynamic properties of different configurations of sticky ends were systematically studied. In their second study, the reaction consisted of DNA tiles similar to our monomers in Fig. 1, with shorter 5-bp sticky end pairs. The most relevant subset of their experiments^58–60^ is the dimerization of rigid QX and DX structures with 2 pairs of sticky ends that are located adjacent to each other. In these variants, the adjusted enthalpy was measured to be 105.1–122.4 kcal mol^–1^. For the entropy of the reaction, they determined the values to be 0.301–0.352 kcal mol^–1^ K^–1^. Together, the free energy of DNA tile dimerization was computed to be 11.8–13.3 kcal mol^–1^, which is 3.2–5.6*RT* higher than our measured free energy.

The relatively-low free energy in our measurement suggests that the sum of inter-molecular penalties between DNA monomers within the nanotubes, which includes the inter molecular strain and electrostatic repulsion, is higher than the energy cost for DX dimers,^60^ QX dimers,^59^ and DNA ribbons.^13^ If we consider Nangreave *et al.*'s DX and QX molecules as simplified models of the association and disassociation of a DNA tile to a growing DNA nanotube, one consideration is that the dimerization process will yield structures with less strain than in our DNA nanotubes. In a related system, strain in tile attachment to a DNA origami seed was inferred to account for a 2 kcal mol^–1^ (or 3.3*RT*) deviation.^23^ Moreover, the close proximity between DNA tiles along the circumference gives rise to higher electrostatic repulsion than in DX dimers, QX dimers, and two-dimensional DNA ribbons. In addition to the inter-molecular strain, the free energy is also dependent on the sticky end sequences. Theoretical Δ*G*° at 37 °C for a random 12-bp DNA helix, using Santa Lucia's nearest neighbor parameters^61^ excluding any stacking energies for the flanking nucleotides, was computed to be 9.9–20.7 kcal mol^–1^ with Δ*G*° = 15.5 ± 1.9 kcal mol^–1^. Interestingly, the Δ*G*° of the sticky end sequences in DNA ribbons, QX, and DX were calculated to be 0.4–2.4 kcal mol^–1^ or 0.7–4*RT* higher than the calculated Δ*G*° for our DNA nanotubes. Together, the sum of higher strain and weaker sticky end is sufficient to explain the relatively low free energy value in our experiments.

Finally, another meaningful value to compute is the melting temperature *T*_m_ of DNA nanotubes. From simple thermodynamics, the melting temperature in Kelvin can be calculated as6
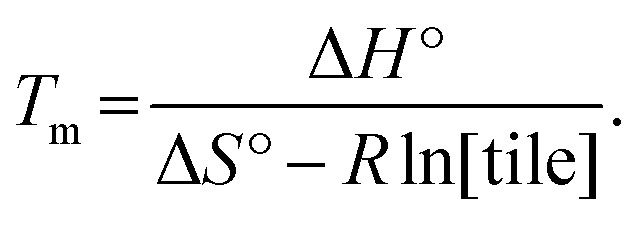



Using the theoretical values from the kTAM,^49^ the melting temperature for a reaction with 100 nM, 200 nM, 300 nM, 400 nM, and 500 nM free tile concentration is calculated to be 43.5 °C, 44.9 °C, 45.7 °C, 46.4 °C, and 46.9 °C, respectively. The calculated values are less than 8 °C higher than the measured equilibrium temperature in the polymerization rate *vs.* temperature plots (Fig. S2†). Similarly, the discrepancy in *T*_m_ is likely because the kTAM number is derived from a simple model and ignores the inter-molecular strain and sequence dependence of Δ*H*° and Δ*S*°. Nonetheless, this relative agreement hints at the usefulness of the simple energetics model in the kTAM for estimating thermodynamic values in DNA self-assembly.

### Comparison with the polymerization rates of actin and microtubules

The kinetic Tile Assembly Model possesses the same kinetic and thermodynamic features as the kinetic model for actin polymerization.^37^ The forward rates of DNA nanotube, actin filament, and microtubule assemblies are modeled as reactions that depend on the free monomer concentration-dependent reaction. Actin filaments and microtubules are asymmetric polymers. The polymer ends have different thermodynamic free energies and kinetic rates. The association rate constant for an ATP bound actin monomer to attach to an actin filament has been measured at the single molecule level to be 0.5 × 10^6^ M^–1^ s^–1^ and 7.4 × 10^6^ M^–1^ s^–1^ for the pointed and the barbed end, respectively.^37^ For microtubules, the association rate constant for α,β-tubulin bound GMP-CPP, an unhydrolyzable analog of GTP, to dock to a microtubule at 37 °C has been measured by bulk assay to be 5.4 × 10^6^ M^–1^ s^–1^.^62^ The association rate constant *k*_on_ values of actin and microtubules are comparable to the measured *k*_on_ in our assay. The monomer dissociation rate for actin and microtubule polymerization depends on the bond strength. The dissociation rate of fuel-bound monomers, such as ATP-actin and GTP-tubulin, is slower than waste-bound monomers. The qualitative and quantitative similarities between the DNA nanotube and actin provide additional support for the DNA nanotube as an attractive engineering material for *de novo* creation of an artificial cytoskeleton.

Although both polymers have comparable *k*_on_ values, typical polymerization of actin and microtubules is on the order of 1 layer per second or faster, compared to the 0.1 layer per second mean polymerization rate of DNA nanotubes reported here. Faster polymerization gives actin and microtubules morphological flexibility. These biopolymers can assemble structures when a cell needs them and stabilize them by capping proteins. The faster cytoskeleton polymerization rate is a direct result of the higher free monomer concentration in cellular *milieu*, which is on the order of 1 μM. In our study, the relatively high spontaneous nucleation rate in DNA nanotubes prevented us from performing polymerization assays at comparable concentrations to those of the actin and microtubules. Hyman *et al.* ^62^ have shown that the coupling between polymerization and stochastic GTP hydrolysis is responsible for the slow spontaneous nucleation rate of protein microtubules. Docking of an α,β-tubulin monomer that is bound to GTP on a growing microtubule, triggers the stochastic GTP hydrolysis reaction, which weakens the tubulin–microtubule binding and increases the dissociation rate significantly. Inspired by this elegant solution, it will be interesting to examine how to incorporate energy consuming reactions into the interaction between DNA tiles and between DNA tiles and DNA nanotubes in order to achieve a higher nucleation barrier than the one observed in the existing passive DNA nanotube system, such as the one used in this work.

## Concluding remarks and outlook

From single-filament movies, we were able to systematically test a mathematical model of DNA self assembly while extracting both the kinetic and thermodynamic parameters of DNA nanotube polymerization. The polymerization model depends on the tile concentrations and is sensitive to reaction temperature. To the best of our knowledge, this experiment is the most accurate measurement of DNA tile-based self-assembly to date. Our experiment justifies the use of polymerization theory developed for one-dimensional cooperative polymers, such as microtubules and actin, to accurately model DNA nanotube polymerization.

Further, we expect that the common core model will be suitable for many types of tile-based self-assembled DNA nanotubes – such as variations on DX tiles,^7,63^ triple-crossover (TX) tiles,^64^ multi-crossover (MX) tiles^65^ and even “4 × 4” tiles^66^ – so long as tile formation is cleanly separated from tube formation, tiles are relatively rigid, a regular lattice is formed, and similar tile assembly pathways arise. It is less clear whether the basic models studied here can be applied without modification to DNA nanotubes that self-assemble directly from single-stranded DNA,^15,67,68^ because the increased potential for flexible interactions between unformed or partially formed oligonucleotides may introduce complications. Synthetic RNA filaments,^69,70^ which may form *via* distinct self-assembly pathways, may also require distinct modeling features.

The most basic demonstration of non-equilibrium polymer dynamics is steady elongation or shortening at a constant monomer concentration that is far from the critical DNA tile concentration. More elaborate non-equilibrium polymer behaviors can be envisioned, such as coupling the DNA nanotube polymerization described in this paper with an analog of nucleotide hydrolysis.^9^ It is conceivable that this biomimetic strategy could potentially recapitulate the more complex non-equilibrium cytoskeleton-based dynamics,^71^ such as treadmilling^72^ and dynamic instability,^73^ where polymerization and depolymerization co-exist at steady state without ever reaching equilibrium. These novel dynamics can only be observed at the single-filament level, as demonstrated with the TIRF assay reported here.
