# Supplementary material for: Thermodynamics and kinetics of DNA nanotube polymerization from single-filament measurements
Source: Chem Sci. 2015 Feb 20;6(4):2252–67. doi: 10.1039/c3sc53331j (PMC5645730; doi:10.1039/c3sc53331j)
Supplement: Supplementary file 1 [file SC-006-C3SC53331J-s001.pdf]

Electronic Supplementary Material (ESI) for Chemical Science.
This journal is © The Royal Society of Chemistry 2015

Thermodynamics and kinetics of DNA nanotube polymerization from single-filament measurements

Supplementary Information

Rizal F. Hariadi,[1] Bernard Yurke,[2] and Erik Winfree[3]

[1]*Applied Physics, California Institute of Technology, Pasadena, California 91125, USA*
present address: Cell and Developmental Biology,
University of Michigan, Ann Arbor, Michigan 48105, USA
[2]*Materials Science and Engineering Department and Electrical and Computer Engineering Department,*
Boise State University, Boise, Idaho 83725, USA
[3]*Bioengineering, California Institute of Technology, Pasadena, California 91125, USA*

$[tile]$ (nM)	N (nanotubes)	T (°C)	k_{on} ($\times 10^5$ /M/sec)	ΔH° (kcal/mol)	ΔS° (kcal/mol/K)
0	162	30.5−41.4	11.9±0.10	72.6±3.1	0.201±0.010
100	55	33.6−41.4	10.0±22.1	143±46	0.424±0.145
200	47	35.2−41.4	5.25±1.24	118±65	0.346±0.208
300	39	36.7−41.4	5.45±2.10	116±84	0.340±0.266
400	26	38.3−41.4	5.25±3.76	112±126	0.328±0.401
500	30	39.8−41.4	3.21±1.01	138±46	0.413±0.149
global fit	347		5.99±0.15	87.9±2.0	0.252±0.006

TABLE S1: Summary of the inferred kinetic and thermodynamic parameters from "local" fits using only data taken at the indicated free monomer concentration (far left column). We consider the global fit (bottom) to be reliable, while the local fits are highly variable and unreliable.

T (°C)	N (nanotubes)	$[tile]$ (nM)	k_{on} ($\times 10^5$ /M/sec)	k_{off} (/sec)	K_d (nM)
33.6	16	0−100	5.90±0.61	0.0145±0.0040	24.8±9.3
35.2	35	0−200	5.06±0.42	0.0168±0.0052	33.7±12.9
36.7	39	0−300	5.12±0.34	0.0478±0.0060	93.5±18.1
38.3	48	0−400	6.64±0.32	0.127±0.008	190±21
39.8	68	0−500	6.21±0.30	0.266±0.010	428±36
41.4	62	0−500	5.52±0.50	0.497±0.016	901±110

TABLE S2: Summary of the inferred reaction rate constants from "local" fits using only data taken at the indicated temperature (far left column). These data are plotted in Fig. 6. The equilibrium dissociation constant K_d was calculated by the ratio between "locally" measured k_{off} and k_{on}.

UV absorbance of DNA nanotube

We chose the reaction temperature range based on the annealing curve for 200 nM Cy3-labeled DNA tiles. The UV absorbance measurement were performed using an AVIV 14DS spectrophotometer (AVIV Biomedical, Lakewood, NJ) equipped with a computer-controlled sample temperature. We informally defined the formation temperature as the temperature at which the slope of the annealing curve suddenly changed due to the formation of DNA nanotube nuclei and elongation of the stable nuclei. Because nucleation is a kinetic phenomena, the measured formation temperature depends on the speed of annealing. In Supp. Fig. S1, the formation temperature was measured to be roughly 35 °C at 0.1 °C temperature step with 15 sec equilibration time. Indeed, we did not observe DNA nanotube nucleation in all 200 nM polymerization experiments at temperatures above 35 °C resulting in valid rate measurements. In contrast, all polymerization data that were taken at 200 nM free monomer concentration at temperature below 35 °C suffers from spontaneous nucleation, consistent with the bulk UV measurement.

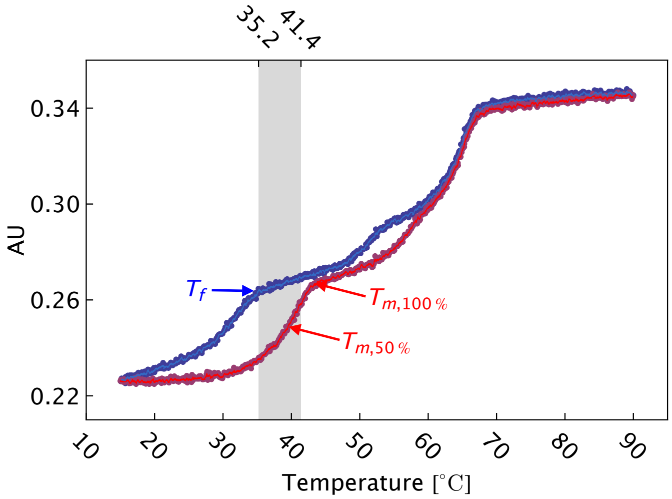

FIG. S1: The annealing (blue) and melting (red) curves of Cy3-labeled DNA nanotubes at 200 nM tile concentration guided the reaction temperature range of this work. The shaded region corresponds to the temperature range where the collected valid measurements of DNA nanotube polymerization at a 200 nM concentration of free tiles. At that tile concentration, DNA nanotube nucleation becomes noticeable at ≈35 °C. In the bottom left plot of Supp. Fig. S2, we were able to acquire valid polymerization rate measurements at this tile concentration down to 35.2 °C (the lower bound of the gray-shaded region), indicating consistency between the UV absorbance and single-molecule microscopy experiments.

The maximum temperature of our assay was set by the safe operation temperature of our objective heater, and not by any insight from the melt experiment or other assay. It is wise to not heat the objective above 50 °C. In this work, we had to heat the objective at 47 °C to achieve a 41.4 °C sample temperature. However, as the DNA nanotube melting temperature is only slightly above 42 °C, in this case, experiments at higher temperatures would not have been interesting.

Thermodynamics analysis.

To examine the temperature dependence of polymerization and depolymerization rates in Fig. S2, we re-plotted the data of Fig. 5 against temperature for each distinct free monomer concentration. For each subpanel, we obtained thermodynamic parameters from a local non-linear fitting with Eqs. 1 and 2. The inferred enthalpy and entropy parameters from the local fits are summarized in Table S1. The k_{on}, ΔH° and ΔS° from local fits were within a factor of 10 for k_{on}) or 2 (for ΔH° and ΔS°) of the global fit values. The local extractions of k_{on}, ΔH° and ΔS° at higher monomer concentrations were progressively less reliable because of the narrower valid temperature range due to rapid nucleation at high free monomer concentrations. Because of the inherent problem in extracting kinetics and thermodynamics parameters from local fitting, we only use the global fitting values of k_{on}, ΔH° and ΔS° elsewhere in the paper.

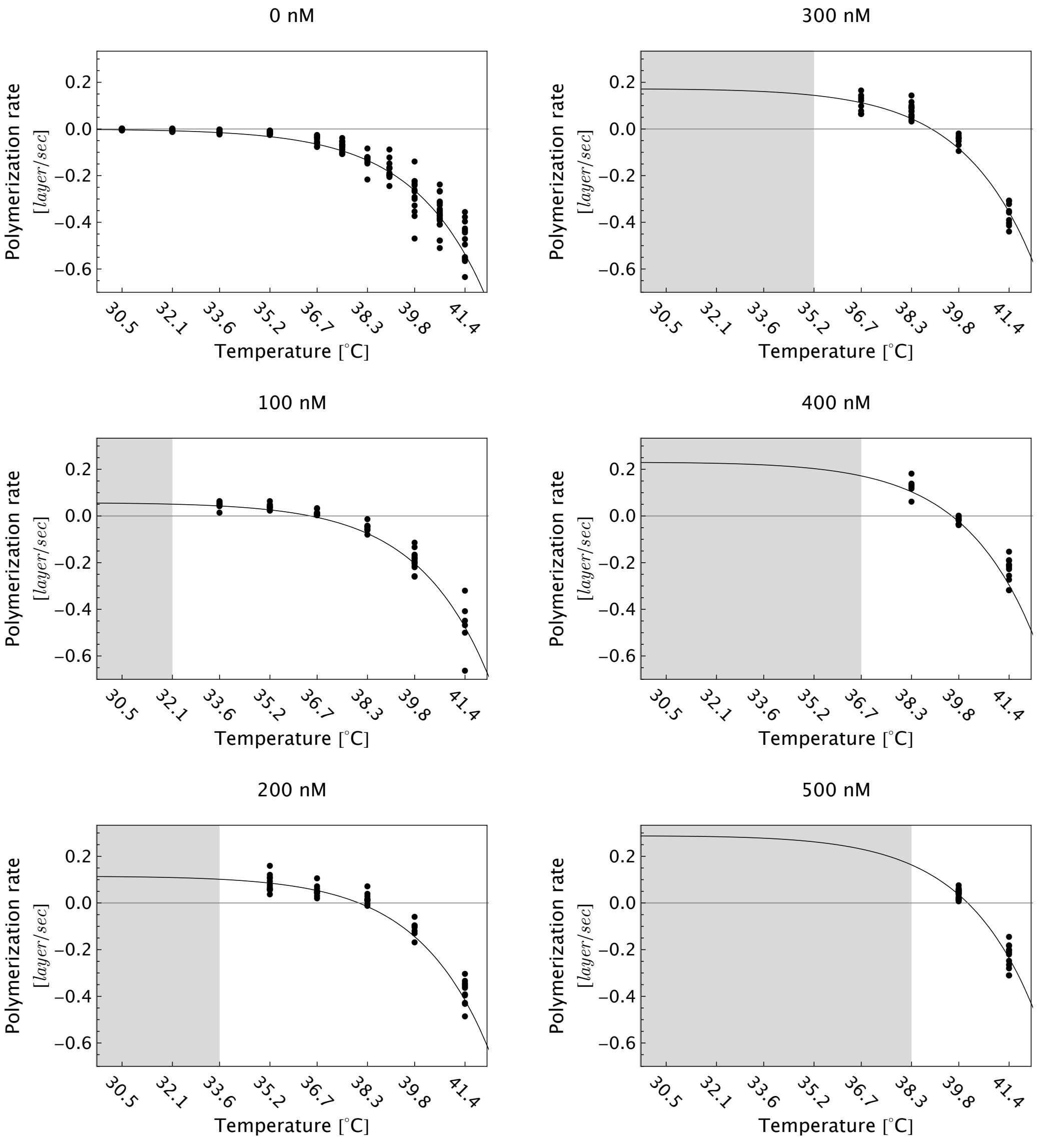

FIG. S2: At a given free tile concentration, DNA nanotubes elongate faster at lower temperatures, which reveals that DNA nanotube polymerization is at least partly enthalpy-driven. The gray-shaded region indicates the concentrations where significant spontaneous nucleation was observed for a particular reaction temperature. The solid line is the result of global fitting with Eq. 1 and 2.

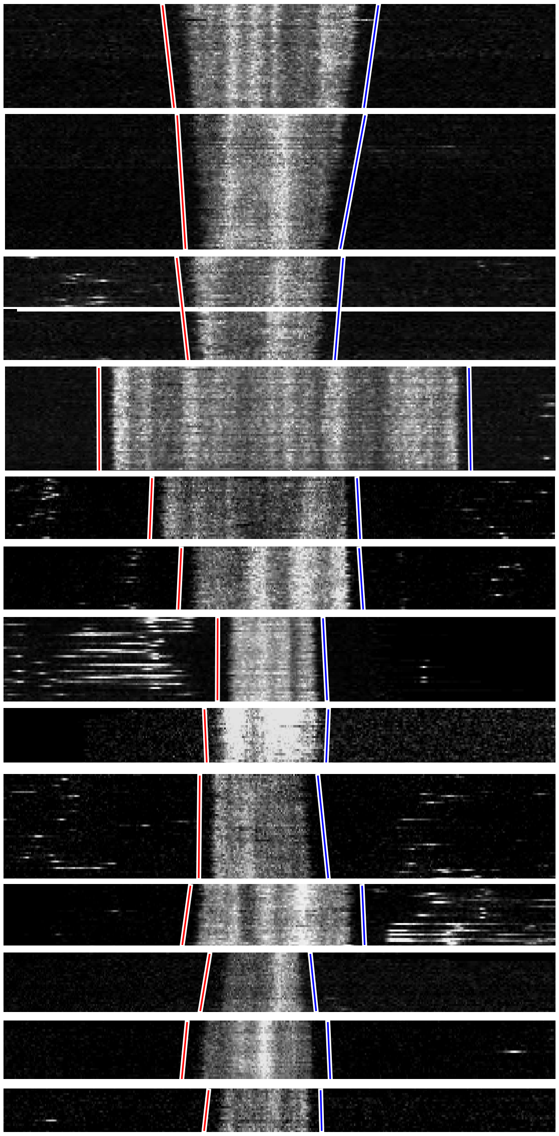

	[tile]	$r_{left} \pm \sigma_{left}$ [layer/min]	$r_{right} \pm \sigma_{right}$ [layer/min]	$\lvert r_{left} - r_{right} \rvert \pm \sqrt{\sigma^2_{left} + \sigma^2_{right}}$ [layer/min]	$\frac{\lvert r_{left} - r_{right} \rvert}{\sqrt{\sigma^2_{left} + \sigma^2_{right}}}$
01	38.3 °C 0 nM	−5.84 ±0.44	−7.28 ±0.58	1.43 ±0.73	1.97
02	38.3 °C 0 nM	−3.53 ±0.74	−10.53 ±1.55	6.99 ±1.71	4.08
03	38.3 °C 0 nM	−5.62 ±0.51	−4.16 ±1.79	1.46 ±1.86	0.78
04	38.3 °C 100 nM	−0.21 ±0.28	0.96 ±0.35	1.18 ±0.45	2.60
05	38.3 °C 200 nM	2.28 ±1.10	2.95 ±1.82	0.67 ±2.13	0.32
06	38.3 °C 200 nM	2.71 ±1.06	3.81 ±2.09	1.10 ±2.34	0.47
07	38.3 °C 300 nM	0.20 ±0.69	2.82 ±1.68	2.62 ±1.82	1.44
08	38.3 °C 300 nM	−2.54 ±2.20	−2.46 ±2.12	0.08 ±3.06	0.03
09	38.3 °C 400 nM	0.58 ±0.50	5.74 ±1.41	5.16 ±1.50	3.44
10	38.3 °C 400 nM	7.96 ±1.18	2.47 ±2.56	5.49 ±2.82	1.95
11	38.3 °C 500 nM	9.55 ±1.97	5.87 ±1.29	3.68 ±2.36	1.56
12	38.3 °C 500 nM	5.47 ±1.28	2.49 ±1.33	2.98 ±1.85	1.61
13	38.3 °C 500 nM	6.55 ±6.55	1.58 ±2.17	4.98 ±6.90	0.72

FIG. S3: (This page and facing page) Kymographs constructed from movies at a variety of temperatures and free monomer tile concentrations (listed at left). The fits for the left-end growth rate and right-end growth rate are shown in red and blue respectively (with positive numbers indicating polymerization and negative numbers indicating depolymerization) along with the standard error of the fit. At the right, the absolute and relative asymmetry is listed. By 'absolute asymmetry', we mean the difference between the left-end rate and the right-end rate. As an error measure for the absolute asymmetry, we treat the errors on the left and right sides as being statistically independent and thus consider the variance to be additive. By 'relative asymmetry', we divide the absolute asymmetry by its standard error, giving a measure of by how much the asymmetry exceeds the noise.

	[tile]	$r_{left} \pm \sigma_{left}$ [layer/min]	kymograph	$r_{right} \pm \sigma_{right}$ [layer/min]	$\lvert r_{left} - r_{right} \rvert \pm \sqrt{\sigma_{left}^2 + \sigma_{right}^2}$ [layer/min]	$\frac{\lvert r_{left} - r_{right} \rvert}{\sqrt{\sigma_{left}^2 + \sigma_{right}^2}}$
14	39.8 °C 0 nM	−12.54 ±0.69	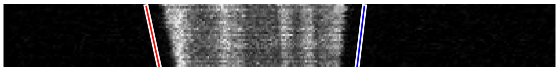	−6.93 ±1.05	5.61 ±1.25	4.47
15	39.8 °C 0 nM	−8.64 ±10.13	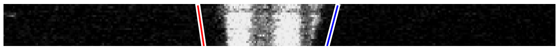	−15.28 ±2.81	6.64 ±10.50	0.63
16	39.8 °C 0 nM	−9.07 ±0.62	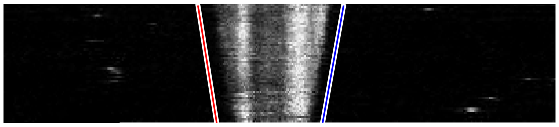	−10.17 ±0.34	1.10 ±0.70	1.57
17	39.8 °C 0 nM	−9.38 ±2.79	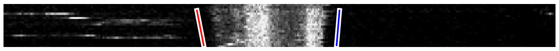	−4.88 ±4.32	4.49 ±5.14	0.87
18	39.8 °C 300 nM	−0.09 ±0.16	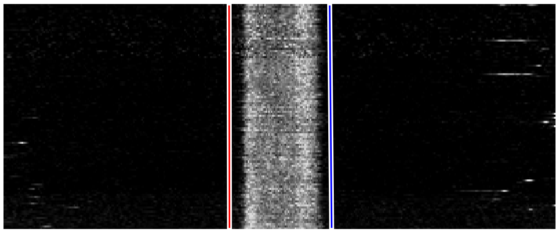	0.50 ±0.15	0.59 ±0.22	2.67
19	39.8 °C 300 nM	0.21 ±0.17	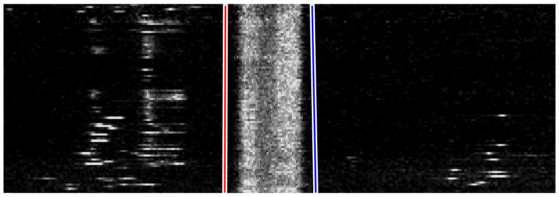	1.08 ±0.27	0.87 ±0.32	2.73
20	39.8 °C 300 nM	0.68 ±0.16	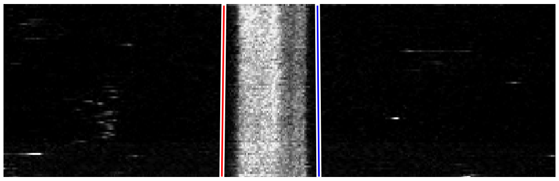	0.48 ±0.16	0.20 ±0.22	0.88
21	39.8 °C 400 nM	0.38 ±0.58	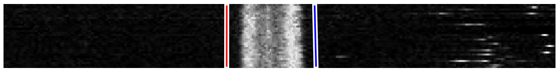	1.33 ±0.69	0.94 ±0.90	1.05
22	39.8 °C 400 nM	1.44 ±0.82	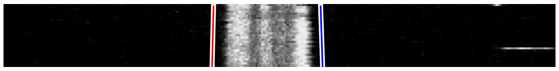	1.90 ±0.71	0.46 ±1.08	0.42
23	39.8 °C 400 nM	0.99 ±0.14	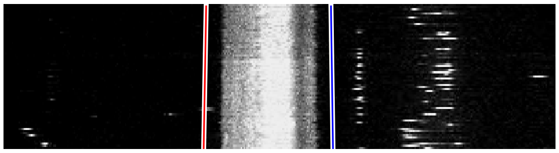	0.80 ±0.20	0.19 ±0.24	0.78
24	39.8 °C 500 nM	6.00 ±0.27	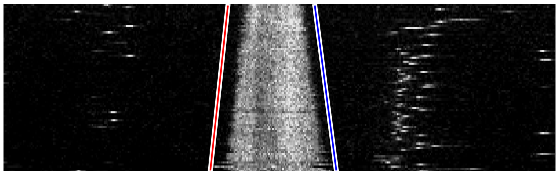	7.36 ±0.27	1.36 ±0.38	3.54
25	39.8 °C 500 nM	4.72 ±0.47	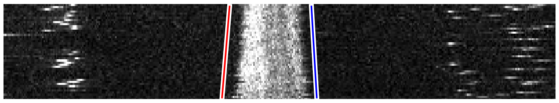	3.36 ±0.61	1.36 ±0.77	1.77

AFM images of opened DNA nanotubes

AFM images were acquired with a tapping mode AFM on a Nanoscope IIIa controller (Veeco Instruments) equipped with nanoAnalytics Q-control III (Asylum Research) under 1× TAE/Mg^{++} (40 mM trisacetate and 1 mM ethylenediaminetetraacetic acid (EDTA) pH 8.3 with 12.5 mM Mg-acetate.4H_2O, pH 8.3) buffer and 110 μm, using an AFM cantilever with a 0.38 N/m spring constant SNL silicon nitride cantilever (Veeco Instruments). The DNA nanotubes were annealed at 1.0 μM tile concentration. Following annealing, the samples were diluted to 100 nM with 1×TAE/Mg^{++}. 10 μL of the diluted sample and 20 μL of 1×TAE/Mg^{++} were deposited on a freshly cleaved piece of mica surface (Ted Pella), approximately 1 cm × 1 cm in size, affixed to a 15 mm diameter magnetic stainless steel puck (Ted Pella) using a hot glue gun. DNA nanotubes spontaneously open on the mica surface, possibly due to the energy gain from the formation of salt bridges between DNA and the mica surface [5]. Under the Mg^{++} buffer, DNA tiles are not completely immobilized and can still attach to or detach from an immobilized opened DNA nanotube. The DNA tile−DNA nanotube interaction effects the validity of circumference measurements of opened DNA nanotubes. To avoid such effect, after 5 minutes, to completely quenched the reaction, 20 μL of 9 mM Ni^{++} in 1×TAE/Mg^{++} was added to the mica puck. The nickel buffer facilitates stronger binding between DNA and mica than the Mg^{++} buffer [2].

We imaged one sample of a pre-formed nuclei stock solution at 1 μm × 1 μm at multiple random coordinates. We used custom-written MATLAB code to flatten the images by subtracting a fitted first order polynomial from each scan line and matching intensity histograms between scan lines. The circumference was measured by counting the number of DNA tiles at 5 random points along the longitudinal-axis of each DNA nanotube. The average value was then used as the circumference of that particular DNA nanotube. Hence, the tabulated DNA nanotube circumference is not restricted to positive integer numbers. A histogram (Fig. 8) was then constructed by binning the data from 1 μm to 15 μm within bin width = 1 μm.

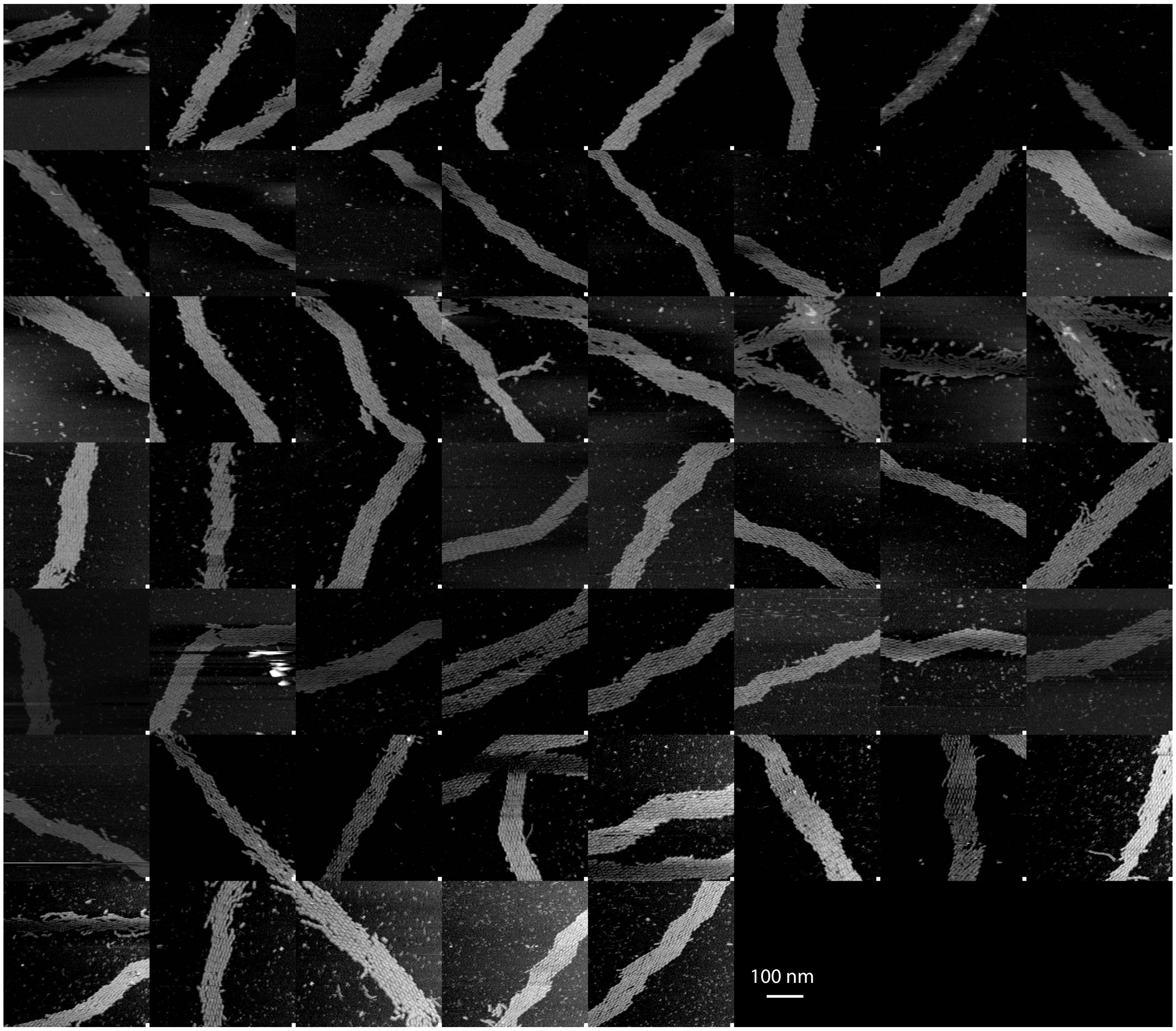

FIG. S4: AFM images of 53 randomly chosen opened DNA nanotubes reveal the circumference heterogeneity of the nuclei. The strong interaction between negatively-charged mica surface, multivalent Ni^{++} ions, and DNA nanotubes leads to the spontaneous DNA nanotube opening. The circumference histogram of DNA nanotube diameter is shown in Fig. 8.

The origin of the asymmetric polymerization

One unresolved issue was the observation of asymmetric polymerization of the two nanotube ends (~~Supp.~~ Fig. S3). The range in measured polymerization rates in a particular field of view was as large as a factor of 3. Local non-specific interaction between the glass surface and the DNA nanotubes is not likely to explain the difference because the local variation of glass surface is minimal and the measured DNA nanotubes were consistently able to diffuse in the confined space close to the glass surface.

Hypothesis 1: DNA nanotubes have an intrinsic polarity that could bias the polymerization rates. – One intriguing hypothesis is that the asymmetry may arise from the physical difference between DNA nanotube ends, such as the location of fluorophores with respect to the attachment site. Since we put the fluorophore at one of the four corners of a DNA tile, only the sticky ends on one end of the DNA nanotubes are decorated with fluorophore. It is also conceivable that the sequence differences between sticky ends 1 and 2* on one end, and 1* and 2 on the other end, could induce different kinetics for attachment and detachment on the two ends.

Hypothesis 2: The asymmetric circumference of the pre-formed DNA nanotube ends gives rise to the observed asymmetric polymerization. – In our experiment, the formation of DNA nuclei involves stochastic end-to-end joining of short DNA nanotube nuclei with either Cy3 or Cy5 fluorophores. These short nanotubes have a range of nanotube circumferences. AFM measurements of opened DNA nanotubes on mica have revealed the circumference distribution of our DNA nanotubes (Fig. 8). Stochastic end-to-end joining of the heterogenous DNA nanotubes would likely generate DNA nanotubes with asymmetric ends.

Hypothesis 3: The observed asymmetric polymerization rates are due to statistical fluctuations and measurement noise, and have no other physical basis. – Due to photon shot noise and other image imperfections, there is statistical error in the identification of tube end positions and alignment of fiducial markers from frame to frame in the kymograph, resulting in statistical error in the estimates of the growth rates. If our estimated level of noise is sufficient by itself to explain the observation of a population of kymographs with highly asymmetric rate measurements, then we would have to conclude that we do not have strong evidence for asymmetric polymerization – whether or not it is actually present.

Hypothesis 4: The asymmetric polymerization was an artifact of our measurement. – The diffusion of DNA nanotubes may have substantial vertical component that can not be captured by two-dimensional imaging. The fluctuation would be random and contribute to the observed asymmetry.

We will address each hypothesis in turn.

Hypothesis 1. Our DNA nanotubes, as described in Fig. 1, are oriented: on one end of the nanotube, which we will call the ‘plus end’, sticky end 1* is labeled with a fluorophore and sticky end 2 is unlabeled; while on the other end of the nanotube, which we will call the ‘minus end’, both sticky ends 1 and 2* are unlabeled. It should first be noted that the thermodynamics for adding a layer of tiles on the plus end must be identical to that for adding a layer of tiles on the minus end. Therefore, at a given free monomer tile concentration and temperature, either growth is thermodynamically favorable on both ends, or growth is thermodynamically unfavorable on both ends. However, given that constraint, the kinetics for both association and dissociation could be different on the two ends. For example, if the fluorophore-labeled sticky end intercalates or binds with the adjacent tile, then an incoming tile will have a slow rate of association – but no such slow-down would occur on the minus end of the nanotube. Analogous interactions could conceivably arise purely due to sequence effects, even in the absence of a fluorophore label.

If there was a significant, systematic difference between the kinetics of the plus and minus ends, then we would expect to see asymmetry in nearly all, rather than just some, of the kymographs. We therefore infer that such kinetic differences are minimal.

Hypothesis 2. We will discuss two ways in which different nanotube circumferences can result in different polymerization (or depolymerization) rates. One effect essentially depends on just the logical of growth front configurations, while the other postulates physical differences that depend on the tube

circumference – both assume that constant-circumference tubes have the same rate at each end.

From a theoretical standpoint, the total ensemble of possible tile configurations along the circumference of DNA nanotubes gives rise to a dependence of the polymerization rate on the nanotube circumference. Based on kinetic arguments, the only attachment site configurations that contribute to the elongation of nanotubes are the ones that can provide two sticky end bonds, $b = 2$. For multi-monomer-wide nanotubes, there are more binding sites in wider nanotubes than in the thinner nanotubes. However, not all of the binding sites can provide $b = 2$ available sticky ends. As examples, the top half of Fig. 7 shows a configuration of 8-monomer wide DNA nanotubes ($m = 8$). The configuration provides 7 sites with $b = 2$, which corresponds to $f_{b=2}^{m=8} = 7/8$. The denominator of the calculated f_b^m is the nanotube circumference and accounts for the larger number of maximum $b = 2$ sites observed in wider nanotubes compared to thinner nanotubes.

The average fraction of sites that can provide $b = 2$ sticky ends per m monomer wide nanotubes $\langle f_{b=2}^m \rangle$ decreases with increasing nanotube circumference m. At the lower limit, $\langle f_{b=2}^{m=1} \rangle$ of a 1-monomer wide nanotube is 1. It is important to realize that a 1-monomer wide nanotube is a chain that behaves differently from nanotubes with $m \geq 2$. The growth of a 1- monomer wide nanotube is isodesmic, which means that the strength of the interactions between monomers in the middle of a 1-monomer wide nanotube is the same as the interaction in the collision between free monomers in solution [1]. It should also be noted that in the analysis of this hypothesis, we are ignoring the circumference-dependent strain energies that are undoubtably experienced by nanotubes [5, 6] and which in this case would probably be relieved by breaking stacking bonds at the nicks adjacent to sticky ends.

For nanotube circumference $m \geq 2$, our model assumed that for m monomer wide nanotubes, the arrangement at nanotube growth front is a one-dimensional random walk with total number of steps equal to $2 \times m$ (Supp. Fig. S5). The factor 2 is based on the number of sticky end pairs (A and B) that serve as binding domains for m monomer wide DNA nanotubes. To form a tubular structure, the numbers of A's and B's have be equal to m. In Supp. Fig. S5, the A and B sticky end interactions are depicted as red (/) and blue (\) lines, respectively. In the language of one-dimensional random walks, a series of A (red /) and B (blue \) lines is equivalent to a sequence of left and right steps of a random walker.

Supp. Fig. S5 shows ensembles of possible tile configurations for the growing end of 1, 2, and 3 monomer wide nanotubes. Each configuration is unique due to the rotational and translational symmetry. The numbers of on-sites and off-sites were calculated by counting the number of peaks and valleys, respectively. The valleys correspond to the attachment sites (on-sites) with 2 available sticky ends. The peaks are the off-site locations where the tile has to break 2 sticky ends to dissociate from the nanotube. Both valleys and peaks are found when the random walk switch direction from A to B, and vice versa. Interestingly, because both A and B have to be present in equal number in all configurations, the number of valleys and peaks also have to be equal.

For finite circumferences m, the number of red and blue steps must be equal, and therefore there are global constraints on what configurations occur, from which we can see that the probabilities cannot be simply those of an unbiased random walk with independent steps. Nonetheless, we show that if nanotube growth occurs according to kTAM rates with every step involving the addition or removal of a single tile that forms exactly 2 sticky end bonds, then the steady-state distribution of random walk configurations (sequences of an equal number of red and blue steps, m each, treating rotationally symmetric states as distinct) must be uniform. Consider a continuous-time discrete-state Markov process on these states, with transitions according to the kTAM. A configuration i that has n valleys and n peaks, will have n neighbors, j, to which it can transition with rate r_f, and from which it can transition with rate $r_{r,2}$, as well as n neighbors, k, to which it can transition with rate $r_{r,2}$ and from which it can transition with rate r_f. Letting p_i be the steady-state probability of configuration i, the dynamics gives

$$\frac{dp_i}{dt} = \sum_j (p_j r_{r,2} - p_i r_f) + \sum_k (p_k r_f - p_i r_{r,2}) \, .$$

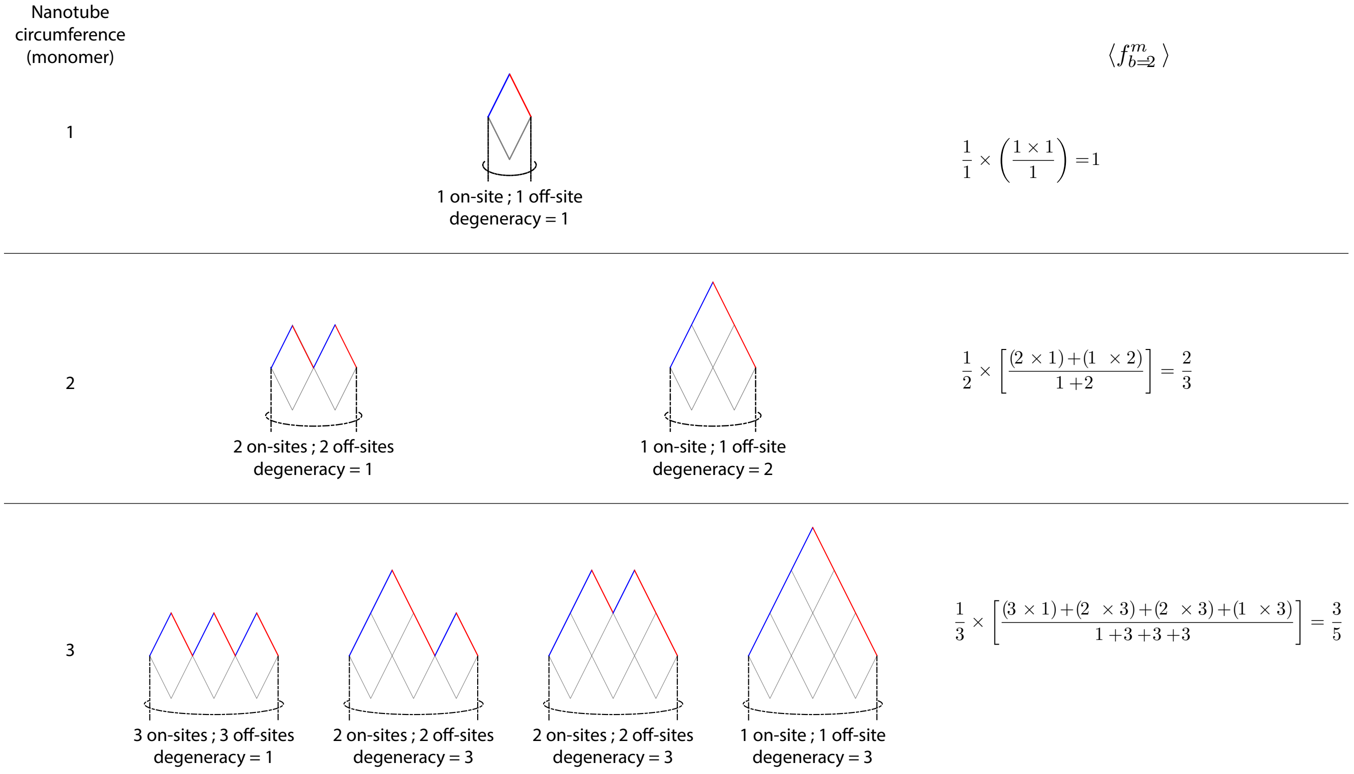

FIG. S5: The possible tile configurations along the nanotube end with circumference = 1, 2, and 3 monomers. A tile is represented as a white diamond. Blue and red solid lines denote the sticky end types. Dashed lines indicate connectivity between two longitudinal edges of the two-dimensional representation of a nanotube end. In all configurations, each tile is required to interact with ≥2 neighboring tiles. The value of $f_{b=2}^{m=2}$ are calculated based on the circumference, numbers of on- and off-sites, and their degeneracies.

Substituting our ansatz that $p_i = 1/N$ where N is the size of the state space, we see that

$$\frac{dp_i}{dt} = n\left(\frac{1}{N} r_{r,2} - \frac{1}{N} r_f\right) + n\left(\frac{1}{N} r_f - \frac{1}{N} r_{r,2}\right) = 0,$$

which establishes that the uniform distribution is the unique steady state for this connected Markov process.

The steady state behavior of nanotubes with m approaching ∞ is less sensitive to the constraints on the random walk. Consequently, $\langle f_{b=2}^{m=\infty} \rangle$ can be estimated from an unconstrained and unbiased one-dimensional random walk. At any position, the probability of finding a valley (on site) or a peak (off site) is $\frac{1}{2}$. Therefore, the limit of $\langle f_{b=2}^{m \to \infty} \rangle$ is $\frac{1}{2}$.

In summary, $\langle f_{b=2}^{m} \rangle$ is maximum for 1-monomer nanotubes, decreases with increasing nanotube circumferences, and approaches $\frac{1}{2}$ as the nanotube circumference approaches infinity. Finally, the kinetic rates of m-monomer wide DNA nanotube polymerization, k_{on}^{m} and k_{off}^{m}, can be effectively estimated from the kinetic rates of interaction for an incoming tile to bind to an available site at the end of a DNA nanotube, k_{on}^{site} and k_{off}^{site}, by the simple expressions $k_{on}^{m} = \langle f_{b=2}^{m} \rangle k_{on}^{site}$ and $k_{off}^{m} = \langle f_{b=2}^{m} \rangle k_{off}^{site}$.

We tested the width-dependent polymerization rate by running stochastic simulations for DNA nanotubes with 1 to 16 monomer circumferences at different free monomer concentrations (Supp. Fig. S6). The rates at which a monomer arrived to an available site at nanotube end, r_f, and disassociated from a non-empty neighboring site, $r_{r,b}$ were computed based on the kTAM. The simulations (Supp. Fig. S6) supported the theoretical dependence of the polymerization rate on the DNA nanotube circumference. Quantitatively, $\langle f_{b=2}^{m} \rangle$ for 1, 2, and 3 monomer nanotubes obtained from the simulations was 1, 0.67, and

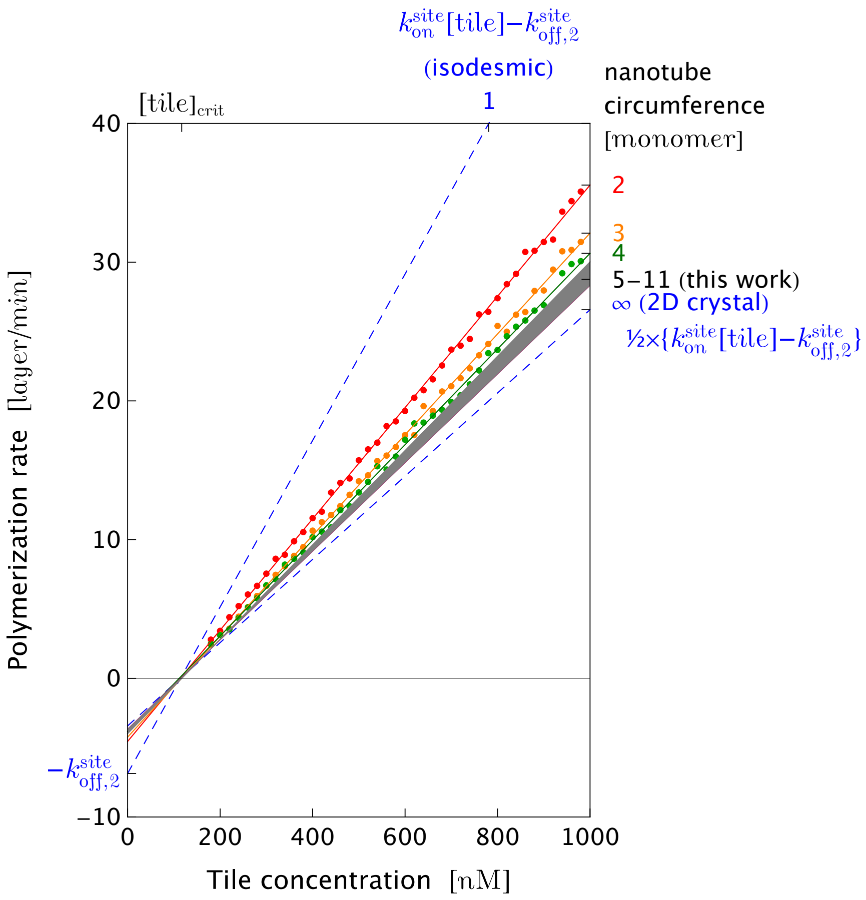

FIG. S6: Stochastic simulation reveals the dependence of polymerization rates on DNA nanotube circumference. Nanotubes with larger circumference polymerize slower than thinner nanotubes. In the plot above, the on rate and the standard free energy of a single sticky end was chosen to be $k_{on}^{site} = 10^6$ /M/sec and $\Delta G^\circ_{se} = 8\ RT$ for all nanotube circumferences. In the plot, $k_{off,2}^{site}$ is defined as the disassociation rate of a DNA tile with 2 bonds and is equal to $k_{on}^{site} e^{-2\Delta G^\circ_{se}/RT+\alpha} \times u_0$ as defined in Eq. 5. The polymerization rates for 2-, 3-, and 4-monomer DNA nanotube are denoted as red, orange, and green circles, respectively. Each data set was subjected to linear fit based on Eq. 3. The simulation results from 5- to 11-monomer nanotubes, which is the circumference range of the DNA nanotubes in our experiment, reside in the gray shaded region. The upper bound (top blue dashed line) is the polymerization rate for 1-monomer DNA nanotubes = $k_{on}^{site}[tile] - k_{off,2}^{site}$, while the lower bound (bottom blue dashed line) is the expected effective rate for the two-dimensional DNA lattice = $1/2 \times \{k_{on}^{site}[tile] - k_{off,2}^{site}\}$.

0.61, respectively, which is in agreement with the theoretical value based on the Markov chain analysis in Supp. Fig. S5 (1, ⅔, and ⅗, respectively).

Because in the model considered here, we ignore any possible circumference-dependent strain energy, i.e. both ΔH° and ΔS° are independent from nanotube circumference, it follows that the on-rates, off-rates, and critical monomer concentration do not depend on the nanotube circumference. Thus, we see that there can be circumference-dependent variability in net polymerization and depolymerization rate constants k_{on} and k_{off} even when (or if) there is no thermodynamic variability for tile attachment and the single-tile association and dissociation rate constants k_{on}^{site} and k_{off}^{site} both remain constant. That said, for the range of nanotube circumferences seen in our experiments (circumferences 5 to 11, Fig. 8), our simulations show that the polymerization rates should not depend strongly on circumference if there is no circumference-dependent strain energy. In fact, simulated polymerization rates for circumferences 5 to 11 were close to the polymerization rate of 2-dimensional DNA lattices (bottom blue dashed line), which was computed to be half the polymerization rate of 1-monomer nanotubes (top blue dashed line).

Since the logical considerations above predicted only small (less than 5%) differences in growth rate for the range of circumferences relevant to this work, it follows that to explain asymmetric growth from end-to-end joined nanotubes of different circumferences, we should turn to explanations involving circumference-dependent changes in the energetics. Based on previous models of DNA nanotubes [5] and two-dimensional DNA lattices [3], the contributions of electrostatic and twist penalty to the overall energetics of DNA nanotubes are expected to depend on nanotube circumference. Indeed, the very fact that a limited range of nanotube circumferences is observed in Fig. 8 suggests that tubes with smaller or larger circumferences are unfavorable thermodynamically, although kinetic effects during tube nucleation surely also play a role in selecting the distribution of nanotube circumferences. Thus, if end-to-end joining occurs (which seems likely) then circumference-dependent thermodynamics and kinetics appears to be a more plausible explanation for asymmetric polymerization rates than the circumference-dependent fraction of available growth sites that was initially discussed.

Hypothesis 3. To test whether statistical variation and measurement error is a sufficient explanation for the observed asymmetric polymerization rates, we performed a simple 'straw-man' test. The straw-man hypothesis is that there is no asymmetric growth, i.e., all observed nanotubes actually grow at equal rates on both ends, but that the measurement error is sufficient to explain the number of apparently asymmetric cases. Because our data was taken at a variety of temperatures and concentrations that each have different average growth rates, the figure of merit that we considered is the difference between the growth rate on the left side and on the right side of the nanotube, normalized by the estimated measurement noise – which we call the 'relative asymmetry' (see Supp. Fig. S3, right column). A histogram of the relative asymmetries that we observed is shown in Supp. Fig. S7. The majority (72%) of kymographs have an asymmetry within 2 standard deviations of zero, which is somewhat less than would be expected if all asymmetry is measurement noise; similarly, the number of events with relative asymmetries larger than 3 is larger than would be expected.

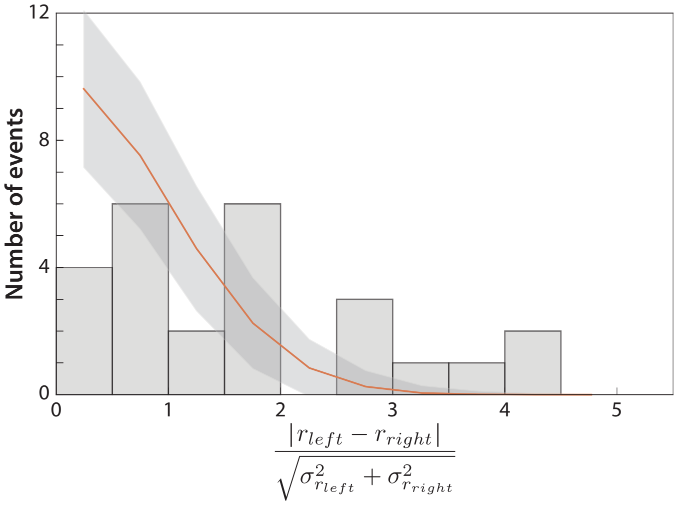

FIG. S7: Histogram of relative asymmetry in our sample of 25 measured kymograph growth rates (bars) and the mean and standard deviation (curves) for a bootstrapped set of 10,000 mock-data histograms, each from 25 mock-samples generated according to the straw-man hypothesis that both ends of each nanotube grow (or shrink) at the same rate, and observed asymmetry is from measurement noise.

As another way to assess what would be statistically expected, we created a bootstrapped sample of straw-man histograms as follows: For each histogram we selected 25 kymographs from the actual data, say $\{(r_{left}, r_{right}, \sigma_{r_{left}}, \sigma_{r_{right}})_i\}_{i=1}^{25}$. Then for each mock-datapoint, we sampled a gaussian using the straw-man hypothesis that the mean and variance of each side of the nanotube should be the same, i.e. $r^*_{left} = \mathcal{N}(\frac{r_{left}+r_{right}}{2}, \frac{\sigma^2_{r_{left}}+\sigma^2_{r_{right}}}{2})$ and, independently, $r^*_{right} = \mathcal{N}(\frac{r_{left}+r_{right}}{2}, \frac{\sigma^2_{r_{left}}+\sigma^2_{r_{right}}}{2})$. Thus, the

mock-standard deviations are $\sigma^*_{r_{left}} = \sigma^*_{r_{right}} = \sqrt{\frac{\sigma^2_{r_{left}}+\sigma^2_{r_{right}}}{2}}$. The mock-relative asymmetry is computed as before, and the mean and variance of the 10,000 mock-histogram bins are shown as the curves in Supp. Fig. S7. (The bootstrapping selection of kymographs was done either with replacement or without, with only infinitesimal differences in the result.) Most bins of the actual data histogram are within two standard deviations of the bootstrapped histograms, with the exception of the most asymmetric bins. We conclude that although most of the observed asymmetry in growth rates can be explained as statistical artifacts, the roughly half-dozen most extreme cases are potentially genuine. This is compatible with occasional end-to-end joining of nanotubes with different circumferences and different tile attachment energetics.

One caveat about the above analysis is that the measurement errors on the left and right sides of each nanotube were assumed to be independent. This is probably not completely true, because some portion of the error comes from imperfect frame-to-frame alignment when constructing the kymographs. Since this type of error moves both ends either too far to the right, or too far to the left, such errors result in correlated noise on the two ends of the nanotube. Sample #25 of Supp. Fig. S3 shows an example where our alignment algorithm did not align the fiducial markers into perfectly vertical lines. In addition to introducing correlations into the left- and right-side errors, imperfect kymograph alignment can directly introduce an asymmetric mean. Although we haven't analyzed such correlations in detail, we expect that they would only quantitatively, and not qualitatively, change the conclusions above.

Hypothesis 4. In the presence of substantial concentration of crowding agent, the diffusion of DNA nanotubes occurs near to the capillary surface. First, based on the excluded volume argument, the two-dimensional diffusion maximizes the entropy of the system. Second, the persistence length of similar DNA nanotubes has been measured to be $\sim 25\ \mu$m [4]. The high persistence length limits the short-scale vertical diffusion of DNA nanotubes. Finally, the decay length of the evanescent wave in our TIRF setup was estimated to be $\sim$100 nm. As a consequence, significant vertical diffusion would generate significant intensity fluctuations in our TIRF images. Kymograph analysis of growing and shrinking DNA nanotubes shows that the variability between different straightened images of a DNA nanotube is very small (Fig. S3).

Movie of anomalous diffusion and side-to-side joining

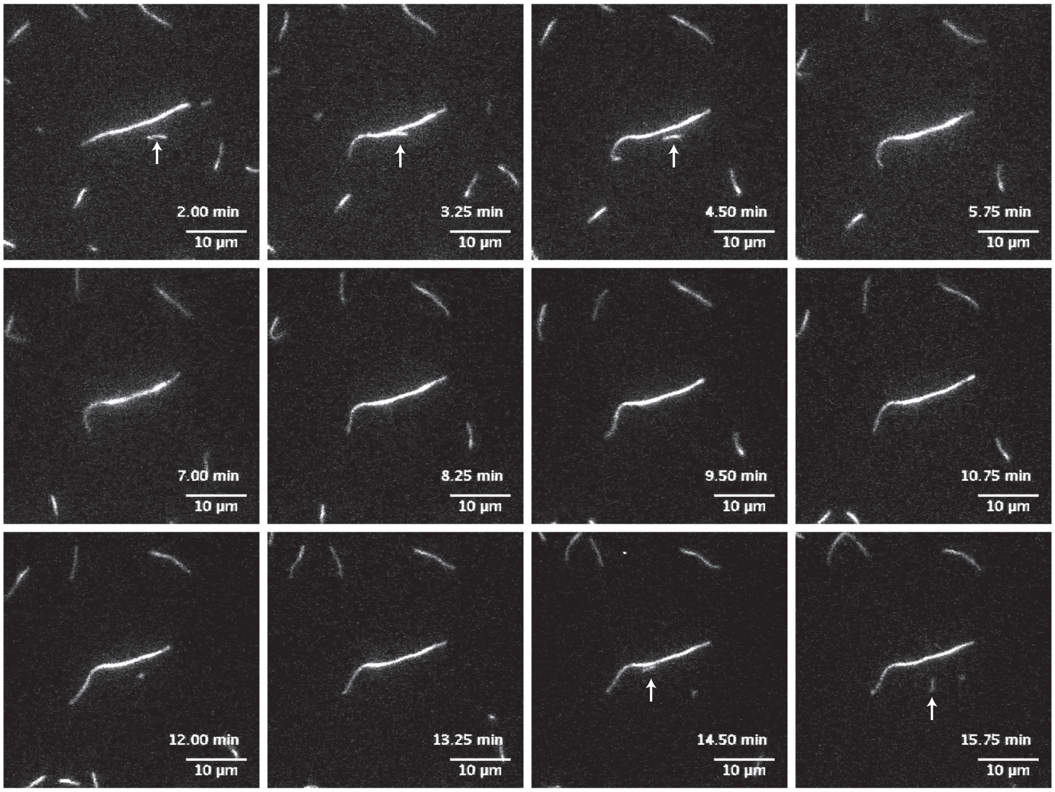

MOVIE S1: TIRF images of mobile DNA nanotubes close the glass surface at 36.7 °C and 0 nM initial free monomer concentration in 1× TAE/Mg^{++} and 0.3% (w/v) methylcellulose. In the presence of the crowding agent, the entropy of the system is maximized when the nanotubes were confined close to a surface, such as the capillary walls. The same entropic force also drives side-to-side joining or "bundling", such as between the short nanotubes and the long nanotubes, as seen near the middle of this movie. After joining, the DNA nanotubes remained mobile relative to each other (length-wide sliding occurred) while they depolymerized from both ends as a physical response to low free monomer concentration. At the end of the movie, the short nanotubes dissociate from the long one, possibly because the entropic gain of the side-to-side joining of short nanotubes is less favorable compared to longer ones.

Movie of complete depolymerization of DNA nanotubes followed by annealing of newly released free DNA tiles

The annealing of free monomers at high concentration nucleated a substantial number of nuclei which immediately elongated and underwent side-to-side joining with other newly-formed nuclei. The total tile concentration was 500 nM and the reaction buffer contains 1× TAE/Mg^{++} and 7.5 mg/mL casein. We used casein to passivate the glass surface and to confine long DNA nanotubes close to the surface. The movie was acquired while the whole microscope room and the sample were initially at an elevated room temperature (37.4 °C). The sample was then melted by increasing the prism temperature to 70 °C. We did not measure the temperature profile of the sample during the spontaneous cooling. The annealing was achieved by simply turning off the prism heater and allowing the sample to spontaneously cool down to 37.4 °C, the elevated room temperature. During this time, the average intensity of DNA nanotube nuclei increased over time, which is the signature of side-to-side joining. Experimentally, the side-to-side joining obscures the elongation of individual DNA nanotube and makes single molecule polymerization measurements unattainable.

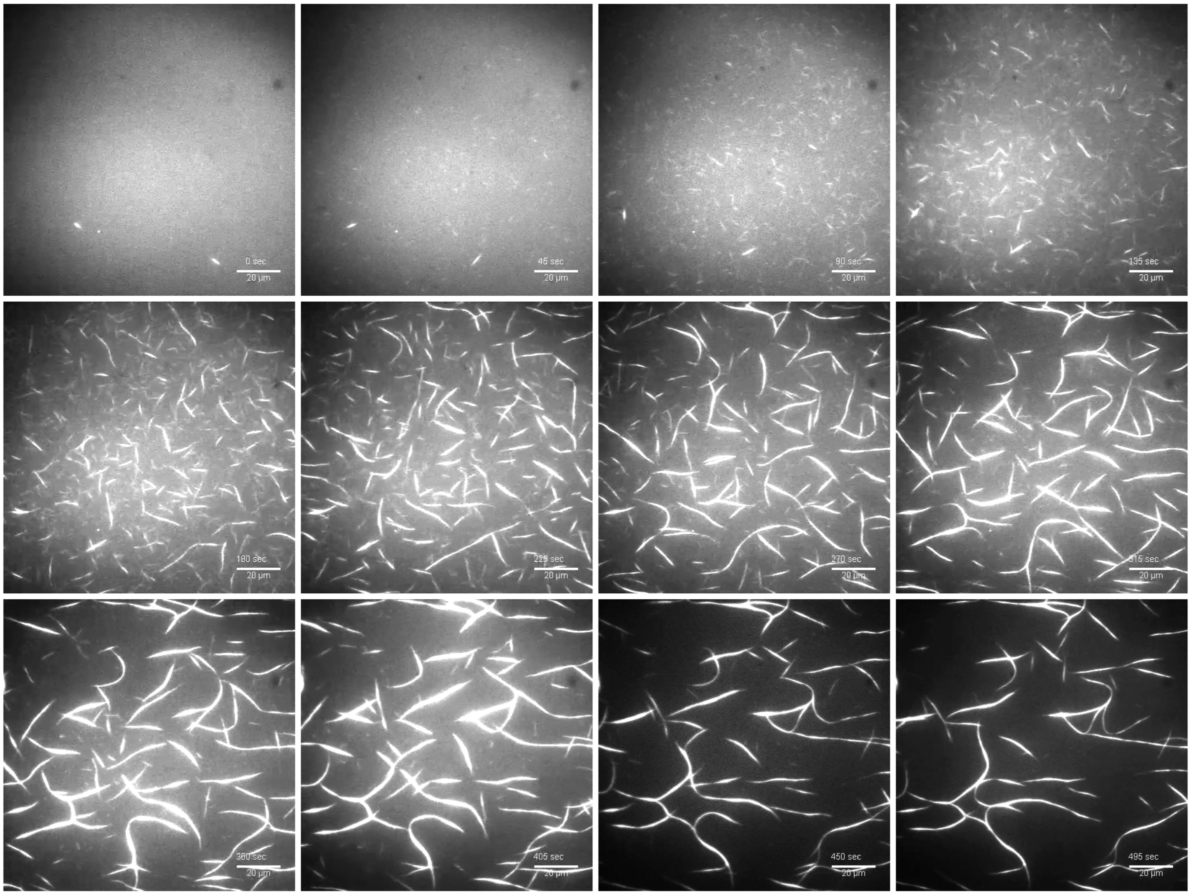

MOVIE S2: In the presence of crowding agent, complete melting of a high concentration of DNA tiles (500 nM) followed by fast annealing produces DNA nanotube "bundles". After the spontaneous nucleation of short nuclei, the newly formed nanotubes "side-to-side" join with each other to maximize the entropy of the crowded environment. Scale bar is 20 μm.

Movie of depolymerization experiment

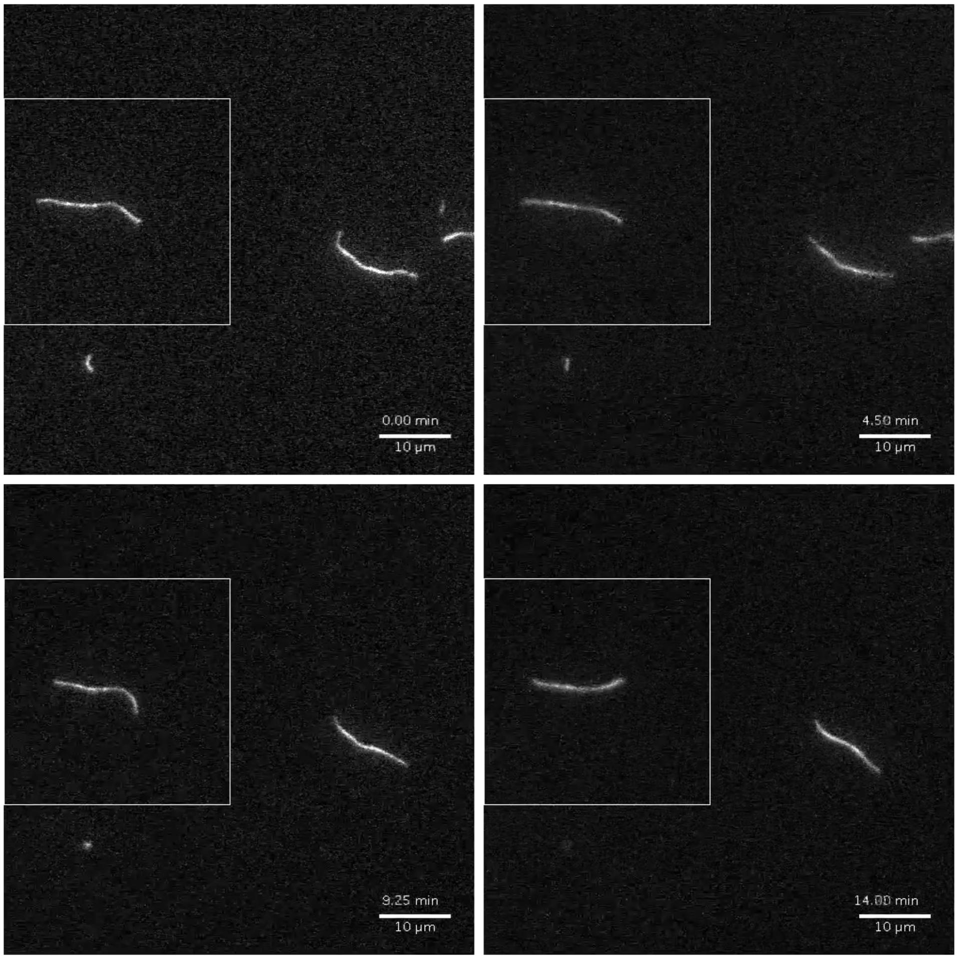

MOVIE S3: DNA nanotubes shrink at 38.3 °C with zero initial monomer concentration and 7 nM initial nuclei concentration, as shown in Fig. 3 of the main text.

Movie of DNA nanotube growth

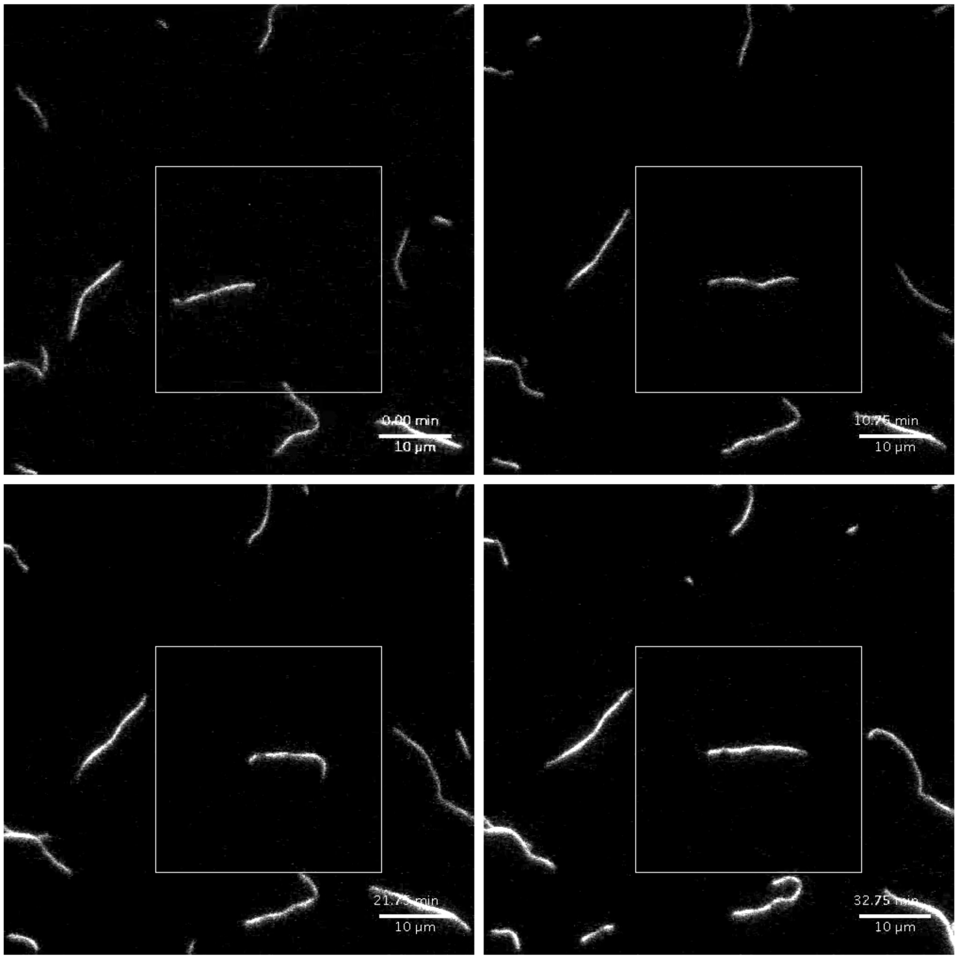

MOVIE S4: DNA nanotubes elongated at 38.3 °C with 400 nM initial monomer concentration and 7 nM initial nuclei concentration, as shown in Fig. 4 of the main text.

[1] Tom F. A. de Greef and E. W. Meijer. Materials science: Supramolecular polymers. *Nature*, 453(7192):171–173, 2008.

[2] Helen G. Hansma and Daniel E. Laney. DNA binding to mica correlates with cationic radius: assay by atomic force microscopy. *Biophysical Journal*, 70(4):1933–1939, 1996.

[3] Rizal F. Hariadi. *Non-equilibrium dynamics of DNA nanotubes.* PhD thesis, California Institute of Technology, March 2011.

[4] Patrick O'Neill, Paul W. K. Rothemund, Ashish Kumar, and Deborah Kuchnir Fygenson. Sturdier DNA nanotubes via ligation. *Nano Letters*, 6(7):1379–1383, 2006.

[5] Paul W. K. Rothemund, Axel Ekani-Nkodo, Nick Papadakis, Ashish Kumar, Deborah Kuchnir Fygenson, and Erik Winfree. Design and characterization of programmable DNA nanotubes. *Journal of the American Chemical Society*, 126(50):16344–16352, 2004.

[6] William B. Sherman and Nadrian C. Seeman. Design of minimally strained nucleic acid nanotubes. *Biophysical Journal*, 90(12):4546–4557, 2006.
